# Rare homozygous cilia gene variants identified in consanguineous congenital heart disease patients

**DOI:** 10.1007/s00439-024-02703-z

**Published:** 2024-09-30

**Authors:** Daniel A. Baird, Hira Mubeen, Canan Doganli, Jasmijn B. Miltenburg, Oskar Kaaber Thomsen, Zafar Ali, Tahir Naveed, Asif ur Rehman, Shahid Mahmood Baig, Søren Tvorup Christensen, Muhammad Farooq, Lars Allan Larsen

**Affiliations:** 1 Department of Cellular and Molecular Medicine, University of Copenhagen, Blegdamsvej 3, 2200 Copenhagen, Denmark; 2 Department of Bioinformatics and Biotechnology, Government College University Faisalabad, Allama Iqbal Road, Faisalabad, 38000 Pakistan; 3 Department of Biotechnology, University of Central Punjab, Lahore, Pakistan; 4 Department of Biology, University of Copenhagen, Copenhagen, Denmark; 5 Centre for Biotechnology and Microbiology, University of Swat, Swat, Pakistan; 6 Rawalpindi Institute of Cardiology, Rawalpindi, Pakistan; 7 Punjab Institute of Cardiology, Lahore, Pakistan; 8 Faculty of Life Sciences, Health Services Academy, Islamabad, Pakistan; 9 Department of Biological and Biomedical Sciences, Aga Khan University, Karachi, Pakistan

## Abstract

Congenital heart defects (CHD) appear in almost one percent of live births. Asian countries have the highest birth prevalence of CHD in the world. Recessive genotypes may represent a CHD risk factor in Asian populations with a high degree of consanguineous marriages. Genetic analysis of consanguineous families may represent a relatively unexplored source for investigating CHD etiology. To obtain insight into the contribution of recessive genotypes in CHD we analysed a cohort of forty-nine Pakistani CHD probands, originating from consanguineous unions. The majority (82%) of patient’s malformations were septal defects. We identified protein altering, rare homozygous variants (RHVs) in the patient’s coding genome by whole exome sequencing. The patients had a median of seven damaging RHVs each, and our analysis revealed a total of 758 RHVs in 693 different genes. By prioritizing these genes based on variant severity, loss-of-function intolerance and specific expression in the developing heart, we identified a set of 23 candidate disease genes. These candidate genes were significantly enriched for genes known to cause heart defects in recessive mouse models (P < 2.4e−06). In addition, we found a significant enrichment of cilia genes in both the initial set of 693 genes (P < 5.4e−04) and the 23 candidate disease genes (P < 5.2e−04). Functional investigation of *ADCY6* in cell- and zebrafish-models verified its role in heart development. Our results confirm a significant role for cilia genes in recessive forms of CHD and suggest important functions of cilia genes in cardiac septation.

**Supplementary Information:**

The online version contains supplementary material available at 10.1007/s00439-024-02703-z.

## Introduction

Congenital heart defects (CHD) comprise simple to complex heart malformations, which affect up to one percent of live births (Liu et al. 2019; van der Linde et al. 2011). The genetics of CHD is complex and monogenic forms of CHD are rare (Morton et al. 2022).

The prevalence of CHD is significantly higher in Asia compared to the rest of the world (Liu et al. 2019). The etiology behind this observation is most likely complex, but many Asian countries have a high degree of consanguineous marriages (Bittles and Black 2010) and the risk of CHD is increased in children of consanguineous couples (Shieh et al. 2012).

Recessive genotypes (RGs) may thus represent a CHD risk factor in some Asian populations, especially in relation to consanguineous marriages.

A limited number of studies have been conducted to identify variants, genes and molecular mechanisms associated with autosomal recessive inherited CHD in the general population. Whole-exome sequencing (WES) analysis of 2645 parent–offspring trios suggested that RGs may account for nearly 2% of CHD (Jin et al. 2017). Genes involved in cilia structure or function (cilia genes) are significantly enriched for RGs in CHD probands, particularly in patients with heterotaxy/laterality defects (Watkins et al. 2019). Cilia are thin microtubule-based antenna-like organelles that project from the surface of most cells (Anvarian et al. 2019). In the developing embryo, cilia play major roles in establishing left–right asymmetry and detecting and conveying cellular signalling, which control organ and tissue development, including the heart (Djenoune et al. 2022; Koefoed et al. 2014). The association between RGs and cilia genes in CHD is corroborated by WES studies of CHD patients with heterotaxy/laterality defects or other severe heart malformations (Dong et al. 2022; Li et al. 2019) and from the result of a recessive forward genetic screening of mice (Li et al. 2015).

Genetic analysis of CHD patients from populations with high frequency of consanguinity represent a potential source for identification of novel CHD disease genes and mechanisms. Pakistan has one of the highest rates of consanguineous marriages in the world, with over 50% of marriages between second cousins or closer relatives (Bittles and Black 2010). In the current project, we used WES to analyze a cohort of Pakistani CHD probands originating from consanguineous unions.

## Methods

### Patients

Forty-nine patients from 48 families were recruited from cardiology departments in Rawalpindi and Lahore, Pakistan. The study followed the declaration of Helsinki and was approved by the Institutional Review Board GC University Faisalabad, Faisalabad, Pakistan. Informed consent was obtained from all participating individuals or their parents for the collection of blood samples, genetic analyses, and publication of genetic information. Patients were diagnosed by transthoracic echocardiography, cardiac catheterization, or surgery.

Consanguinity was confirmed by pedigree analysis after constructing pedigrees of each family, based on information from family elders.

### Whole exome sequencing

Blood samples were collected from patients with an ASD and DNA was extracted using a Puregene Blood Core Kit C (Qiagen, Aarhus, Denmark). Whole exome sequencing (WES) was performed by BGI Genomics (Shenzhen, China). Coding DNA was captured using Agilent V6(60 M) capture library and sequenced using PE100 sequencing on BGISEQ sequencing platforms. Sequencing-derived raw image files were processed by BGISEQ basecalling Software with default parameters and the sequence data of each individual was generated as paired-end reads and stored in FASTQ format. Clean data was produced by data filtering on raw data. All clean data of each sample were mapped to the human reference genome (hg19) using Burrows-Wheeler Aligner (BWA V0.7.15) (Li and Durbin 2010). Variant calling was performed following recommended Best Practices for variant analysis with the Genome Analysis Toolkit (GATK) (Van der Auwera et al. 2013). Local realignment around InDels and base quality score recalibration were performed using GATK v3.7 (DePristo et al. 2011; McKenna et al. 2010), with duplicate reads removed by Picard tools v2.5.0 (http://broadinstitute.github.io/picard/). All genomic variations, including SNPs and InDels were detected using GATK. Variant annotation was performed using the SnpEff tool (Cingolani et al. 2012). An average of 90,345,572 clean reads (9027.12 Mb) were obtained, with an average CG content of 54%. Mean sequencing depth of target regions was 106.1x. On average, per sample, 99.8% of targeted bases were covered by at least 1 × coverage and 97.4% of the targeted bases had at least 10 × coverage.

### Variant filtering

Only homozygous variants with ≥ 10 × coverage were included. Rare homozygous variants (RHVs) were identified according to minor allele frequency (MAF) in public databases; MAF < 0.01 in 1000 genomes (total population), GnomAD total population and GnomAD south Asian population, respectively, and MAF < 0.02 among the 49 patient samples. In addition, we filtered for protein altering variants (PAVs), by removing synonymous variants, intron variants (except splice region variants) and intergenic variants.

### Identification of regions of homozygosity

Regions of homozygosity were identified from vcf files using HomozygosityMapper (Seelow and Schuelke 2012). Setting were adjusted for identification of blocks of at least 50 homozygous variants. Circa software v.1.2.2 (OMGenomics, Redwood City, CA) was used to visualize the regions of homozygosity identified using Homozygocitymapper.

### Cilia localization of ADCY6 during cardiomyogenesis

Temporal localization of ADCY6 to the primary cilium during in vitro cardiomyogenesis was evaluated by immunofluorescence microscopy analysis (IFM) in cultures of mouse embryonic P19.CL6 stem cells, which were induced to differentiate into cardiomyocytes as previously described (Clement et al. 2009b). Cells were fixed in 4% paraformaldehyde and permeabilized in 0.2% Triton X-100. Blocking was performed using 2% bovine serum albumin (BSA), and cells were incubated in primary antibodies (mouse anti-acetylated α-tubulin [1:2000, Sigma-Aldrich T6793], ADCY6 [1:500, Invitrogen PA5-118931]) overnight at 4 °C. Secondary antibodies were incubated for 45 min, and 4′,6-diamidino-2-phenylindole (DAPI) staining performed prior to mounting. Cardiomyogenesis was validated by IFM using the following primary antibodies: mouse anti-SOX2 [1:400, R&D Systems, MAB2018], goat anti-GATA4 [1:200, Santa Cruz, Sc-1237], mouse anti-α-actinin [1:400, Sigma-Aldrich, A7811] and rabbit anti-Troponin-I [1:400, R&D Systems, MAB8594]. Secondary antibodies: Alexa-Fluor^568^-conjugated donkey anti-mouse, Alexa-Fluor^568^-conjugated donkey anti-rabbit, Alexa Fluor^488^-conjugated donkey anti-mouse, Alexa-Fluor^488^-conjugated donkey anti-rabbit, Alexa Fluor^488^-conjugated donkey anti-goat IgG [all 1:600, Invitrogen/Life Technologies].

Fluorescence images were captured on a fully motorized Olympus BX63 upright microscope with an Olympus DP72 color, 12.8-megapixel, 4140 × 3096-resolution camera. The software used was Olympus CellSens Dimension version 1.7, which was able to do deconvolution, 3D isosurface projections on captured z stacks and slice views. Images were processed for publication using Image J version 2.0 and Adobe Photoshop CS6 version 13.0. For quantifications, the mean fluorescence values for ADCY6 at the cilium-centrosome axis were set relative to the fluorescence values in background areas of the cytosol. All data were gathered for n = 3, statistical calculations were performed with the ANOVA test and data were presented as violin plots using GraphPad Prism 9 software.

### Zebrafish husbandry

The zebrafish AB Wild-type (WT) strain was obtained from the Zebrafish International Resource Center (ZIRC). All animals were maintained in the animal facility at the University of Copenhagen, Denmark. WT zebrafish were raised in a constant light–dark cycle at 28 °C according to standard protocols. Staging and maintenance of embryos were carried out as previously described (Kimmel et al. 1995). All experiments were approved and conducted according to licenses and guidelines from the Danish Animal Experiments Inspectorate (Protocol code: P20-387).

### crRNA design and selection

crRNAs were designed for *adcy6a* (ENSDARG00000061445) and *adcy6b* (ENSDARG00000027797) and evaluated using online tools from IDT (Integrated DNA Technologies)(https://eu.idtdna.com/site/order/designtool/index/CRISPR_CUSTOM), CCTOP (Stemmer et al. 2015) (https://cctop.cos.uni-heidelberg.de:8043), and CRISPOR (Concordet and Haeussler 2018) (http://crispor.tefor.net). The designing of each crRNA was done according to previously described protocols (Kroll et al. 2021). Briefly, four crRNAs were designed for each *adcy6* orthologue to increase the probability of introducing biallelic frameshift mutations. The four crRNAs were designed, where possible, to target distinct asymmetrical exons whilst avoiding the first exon. Selection of crRNAs was based on the ranking of predicted on- and off-target scores. Designed guides, IDT design codes, and exons targeted for *adcy6a* and *adcy6b* are shown in Supplementary Table S3 and Fig. S8a. Guides were tested for activity before experiments were conducted (Table S4 and Fig. S8b).

### gRNA/Cas9 complex assembly

Assembly of the gRNA/Cas9 complex for injection was performed as described previously (Kroll et al. 2021). In brief, equimolar volumes of crRNAs and tracrRNA were mixed in nuclease-free Duplex buffer (IDT) and incubated at 95 °C for 5 min to create a 57 µM crRNA:tracrRNA complex. Alt.R S.p. HiFi Cas9 Nuclease V3, 61 µM (IDT) was diluted to 57 µM with Cas9 buffer (20 mM Tris–HCl, 600 mM KCl, 20% glycerol). Equimolar volumes of crRNA:tracrRNA and diluted Cas9 were mixed and incubated at 37 °C for 5 min to formulate a 28.5 µM RNP. For each *adcy6* orthologue, the four RNP solutions were mixed in equal volumes and stored at – 20 °C. The same procedure was completed with three *scrambled* crRNAs (Alt-R CRISPR-Cas9 Negative Control crRNA #1, #2, #3, IDT) used as negative controls.

### Injections, genotyping and phenotyping

Approximately 1 nL (28.5 fmol) of pooled RNP was injected into the cytoplasm of single-cell staged embryos. Survival of injected embryos were monitored several hours after injections and 1 day post fertilization (dpf). Lateral images of 2 dpf zebrafish were taken by positioning larvae with 3% methylcellulose under a Zeiss AxioZoom V16 microscope (Carl Zeiss, Brock Michelsen A/S, Denmark). Individual larvae were genotyped accordingly until it was confirmed that crispant mutants were generated in > 95% of the larvae. After confirmation, larvae were genotyped in batches of 10 larvae.

### ISH in zebrafish

Anti-sense *myl7* riboprobes were synthesized from a pGEM-T easy vector before being linearized, digoxygenin (DIG)-labeled, and transcribed with T7 RNA polymerase (Roche). ISH was performed as previously described (Thisse and Thisse 2008) with minor modifications. Briefly, 2 dpf larvae were fixed in both 4% PFA and 100% methanol. Larvae were rehydrated in a series of dilutions and permeabilized with Proteinase K (10 µg/ml) before being re-fixed in 4% PFA and pre-hybridized. Larvae were hybridized overnight at 70 °C with the *myl7* probe. Following washes, larvae were incubated overnight at 4 °C in anti-DIG antibody (1:6000, Roche) before being washed again. Probe staining was detected with NBT/BCIP (Roche) solution. Stained larvae were imaged with a Zeiss AxioZoom 16 microscope and analyzed. Larvae were subsequently genotyped as previously stated.

### Heart rate analysis in larvae

Larvae at 2 dpf were removed from a 28 °C incubator in batches of five and allowed to acclimatize to room temperature. Larvae were anaesthetized with MS222 (1:100, Sigma-Aldrich) for 3 min. Positioning of the larvae in a brightfield microscope was done so that the heart was clearly visible. The heart rate for 15 s was then manually counted. Heart rates for one minute were subsequently calculated and larvae were genotyped as previously stated.

### Statistical analyses

Statistical analyses were performed using Graphpad Prism v.9.5. For data in Fig. 3E and Supplementary Fig. S4, a Mann–Whitney *U* test was performed to obtain exact P values ( https://www.statskingdom.com/170median_mann_whitney.html). Adjustment for multiple testing was performed with the Benjamini–Hochberg method (https://tools.carbocation.com/FDR). Enrichment of known CHD genes was determined by calculating overlap between gene lists. Significant overlap was calculated using hypergeometric statistics (http://nemates.org/MA/progs/overlap_stats.html). A representation factor was calculated as the number of overlaping genes, divided by the expected number of overlapping genes drawn from two independent groups; RF = x/((n*D)/N), where x = number of overlapping genes, n = genes in group 1, D = genes in group 2, N = genes in genome (20,000).

Data from zebrafish experiments are presented as the mean ± SEM. All results from the experiments were validated by independent repetitions and were carried out with n ≥ 3. Representative images and graphs are presented. Data was tested for Gaussian distribution with a Shapiro–Wilk test. Statistical difference between groups was completed by either ordinary one-way analysis of variance, ordinary two-way analysis of variance or with a Kruskal–Wallis test.

A type 1 error level of 0.05 was used in all statistical tests.

## Results

We analysed 49 CHD patients (20 females, 29 males) from 48 consanguineous Pakistani families (Fig. S1). Parents of the patients did not have CHD. Age of the patients ranged from 1 to 53 years. None of the patients had extracardiac defects or heterotaxy and none of the patients had previously been diagnosed with a genetic disorder. Several patients had a history of respiratory symptoms (Table S1) but none of the patients had been diagnosed with Primary Cilia Dyskinesia (PCD). The CHD diagnoses covered VSD (51.0%), ASD (20.4%), TOF (12.2%) and others (16%) (Tables 1, S1).

**Table 1:** Clinical characteristics of patients

Variables	N (%)
Sex	
Female (%)	20 (40.8)
Male (%)	29 (59.2)
Total	49 (100)
CHD diagnosis	
Ventricular septal defect (VSD)	25 (51.0)
Atrial septal defect (ASD)	10 (20.4)
Tetralogy of Fallot (TOF)	6 (12.2)
ASD, pulmonary stenosis (ASD, PS)	1 (2.0)
Atrioventricular septal defect (AVSD)	1 (2.0)
Bicuspid aortic valve (BAV)	1 (2.0)
Mitral valve regurgitation (MVR)	1 (2.0)
Patent ductus arteriosus (PDA)	1 (2.0)
Transposition of the great arteries (TGA)	1 (2.0)
Univentricular heart, TGA	1 (2.0)
VSD, PS	1 (2.0)
Total	49 (100)

None of the patients had extracardiac defects or heterotaxy and none of the patients had previously been diagnosed with a genetic disorder. Detailed clinical characteristics are listed in Table S1

**Fig. 1:**
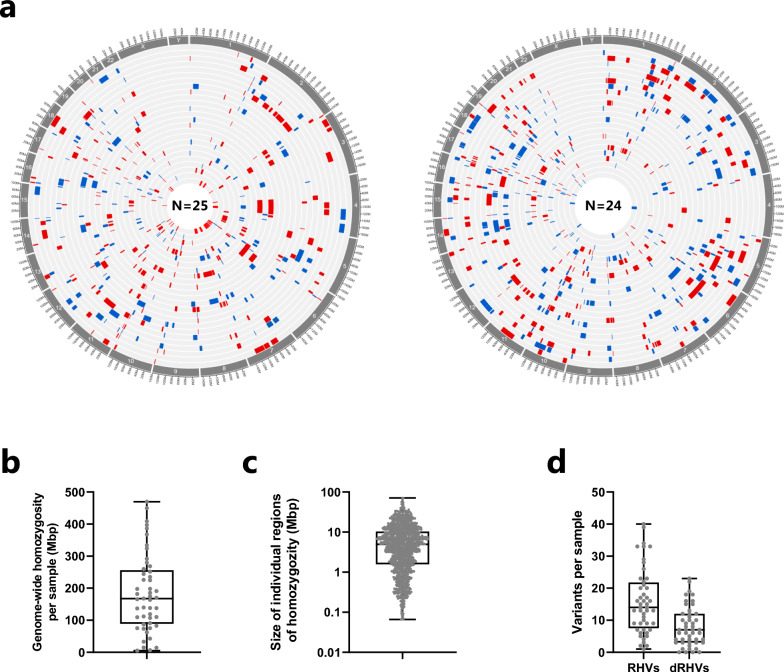
Distribution of rare homozygous variants in CHD patients from consanguineous families. **a** Genomic regions of homozygosity (ROH) in 49 patients. The genomic localization of ROH, containing at least 50 homozygous variants, is shown with red or blue bars. Each circle represents the genome of one patient. The size of each ROH is shown in (C). **a** Total size of ROH per patient in mega base-pairs (Mbp). **c** The size distribution of all 1,167 ROH identified in patients. **d** The number of rare homozygous variants (RHVs) and damaging RHVs (dRHVs) identified per sample

We identified coding variants by whole exome sequencing (WES). Analysis of WES data revealed large regions of homozygosity-by-descent (HBD) in the patient’s genome (Fig. 1a). The genome-wide size of HBD ranged from 5.1 to 469.7 Mbp per sample, with a median value of 167.9 Mbp (5.6% of the genome) (Fig. 1b). We identified a total of 1,168 HBD regions, each encompassing at least 50 homozygous variants. Individual HBD regions ranged from 66.1 Kbp to 71.2 Mbp in size, with a median value of 4.96 Mbp (Fig. 1c). The longest HBD segment per sample was 30.2 Mbp ± 14.8.

By variant filtering, we identified a total of 758 protein altering rare homozygous variants (RHVs) in 693 candidate disease genes (gene-set 1, GS1) (Table S2). Thirty-one (4.1%) of the variants were protein truncating (nonsense or frameshift variants) and 695 (91.7%) of the variants were missense variants. The number of RHVs per sample ranged from one to 40 (median value of 14). The number of damaging RHVs (CADD score ≥ 21) per sample ranged from zero to 23 (median value of 7, Fig. 1d).

We hypothesized that if RHVs are causative of CHD in our patient cohort, we would expect that GS1 was enriched for known CHD genes. To test this hypothesis, we calculated the overlap between the 693 genes in GS1 and curated lists of genes known to cause CHD in mouse models and patients (Table S3) (Audain et al. 2021; Izarzugaza et al. 2020). We observed significant enrichment of human genes causing biallelic, recessive, CHD (RF = 2.3, P = 0.019, Fig. S2) and genes from mouse models of CHD (MmCHD), of which most are recessive models (RF = 1.4, P = 0.023, Fig. S2), but we observed no enrichment of human genes causing monoallelic, dominant, CHD (RF = 0.9, P = 0.471, Fig. S2). The enrichment of genes known to cause recessive CHD in GS1 support that rare RHVs are associated with CHD in our patient cohort.

**Fig. 2:**
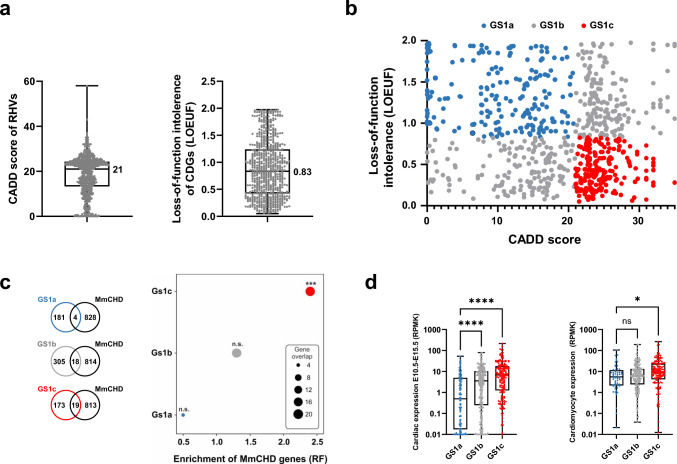
Prioritization of candidate disease genes. **a** Distribution of CADD scores of RHVs identified in the patients (left) and distribution of loss-of-function intollerence score (LOEUF) in candidate disease genes (CDGs, right). Median values are indicated. **b** X–Y plot of 678 CDGs containg rare homozygous variants (geneset 1, GS1). The genes are plottet according to CADD score of RHVs identified in each gene (X-axis) and loss-of-function intolerence of the gene (LOEUF, Y-axis). GS1 was divided in three sub-groups. Low-likelihood CDGs (GS1a, blue): CADD < 21, LOEUF > 0.83. Medium likelihood CDGs (GS1b, grey): CADD < 21 and LOEUF ≤ 0.83 or CADD ≥ 21 and LOEUF > 0.83. High-likelihood CDGs (GS1c, red): CADD ≥ 21 and LOEUF ≤ 0.83. **c** Enrichment of MmCHD genes among three CDG subgroups. Enrichment was calculated by comparing gene-overlap between MmCHD genes and the three sub-groups of GS1. Enrichment is shown as representation factor (RF). A hypergeometric distribution was used to test the significance of the overlaps. **d** Expression level of the three GS1 subsets in mouse embryonic hearts (left) (Cardoso-Moreira et al. 2019) and in vitro cultures of cardiomyocytes (right) (Wamstad et al. 2012). Difference between medians were determined using ANOVA (Kruskal–Wallis test). Asterisks indicate P values: * P < 0.05, *** P < 0.001, **** P < 0.0001. ns: not significant

A high CADD score of a given variant indicates that the variant is more likely to be deleterious to the gene product (Kircher et al. 2014), while a low LOEUF score indicates that a gene is less tolerant to loss-of-function (Karczewski et al. 2020). Thus, as a measure of severity of the RHVs and the intolerance towards loss-of-function, we determined the CADD score of each variant and the LOEUF score of each gene in GS1. The distribution of CADD scores and LOEUF scores of RHVs and GS1 genes, respectively are shown in Fig. 2a. We used the median value of the CADD scores (21) and LOEUF scores (0.83), as cut-off for variant severity and loss-of-function intolerance, and thus define variants with CADD score ≥ 21 as likely damaging and genes with LOEUF ≤ 0.83 as likely intolerant to loss-of-function. Next, we grouped GS1 into three subsets: 185 genes with low likelihood of causing CHD (GS1a, CADD < 21, LOEUF > 0.83), 323 genes with medium likelihood of causing CHD (GS1b, CADD ≥ 21, LOEUF > 0.83 and CADD < 21, LOEUF ≤ 0.83) and 192 genes with high likelihood of causing CHD (GS1c, CADD ≥ 21, LOEUF ≤ 0.83). The CADD score and LOEUF of the three gene subsets are plotted in Fig. 2b and listed in Table S2.

To validate the categorization of GS1 into three gene-sets based on their likelihood of causing CHD, we calculated the enrichment of MmCHD genes (Table S3) within each GS1 subset and observed that only the GS1c subset is significantly enriched for such genes (RF = 2.4, P = 4.3e−04, Fig. 2c). In addition, we performed the same enrichment analysis using a human gene-set obtained from clinical exome sequencing (CES) of CHD patients from a consanguineous cohort (Table S3) (Monies et al. 2019). Analysis of the much smaller human gene-set gave similar results, with enrichment for the GS1c subset only (RF = 8.3, P = 0.001, Fig. S3). Finally, we utilized publicly available transcriptomic datasets (Cardoso-Moreira et al. 2019; Wamstad et al. 2012) to compare the transcription level of genes in mouse E10.5-E15.5 embryonic hearts and cardiomyocytes differentiated from mESC, in each GS1 gene-set (Fig. 2d). For both data materials, the median expression value of GS1c was significantly higher than the median value of GS1a, while the median value of GS1b displayed an intermediate level of expression, compared to the other groups. Based on these analyses, we conclude that GS1c is enriched for CHD disease genes.

We performed a final prioritization of the 192 genes in GS1c by examining their specific expression in the developing heart. To this end, we used RNAseq data from mice (E10.5–E18.5) to compare gene expression in the heart with gene expression in brain (Br), liver (Li) and kidney (Ki). We identified 23 genes with at least two-fold higher expression in the heart compared to the other three tissues during development (Fig. S4) (genes and annotated variants (Richards et al. 2015) are listed in Table 2). These 23 genes are enriched for MmCHD genes (RF = 8.4, P < 2.4e−06) and represent a list of high-likelihood candidate disease genes (CDGs) for CHD in our probands.

**Table 2:** Rare homozygous variants identified in 23 candidate disease genes

Gene	Variant (hg19)	Protein change	MAF^a^	MAF (SA)^b^	CADD score	LOEUF^c^	AD (REF;ALT)^d^	ClinVar^f^	ACMG^g^
*ABLIM1*	10–116213233-G-A	P484L	1.3E−04	4.9E−04	25.3	0.326	0;32	n/a	VUS (PS4)
*ADCY6*	12–49,176,512-A-G	S236P	9.9E−05	7.7E−04	21.4	0.442	0;291	VUS	VUS (PS4)
*CACNA1H*	16–1,254,361-A-T	K785M	3.6E−03	1.9E−03	23.8	0.504	0;88	Benign	VUS (PS4, PP3)
*CACNA1S*	1–201,022,621-C-T	R1254Q	6.6E−04	4.3E−03	32	0.521	0;222	VUS/Benign	VUS (PP3)
*CLASP1*	2–122,120,842-C-G	R1371T	1.6E−04	1.3E−03	24	0.176	0;76	n/a	VUS (PS4)
*DCN*	12–91,572,244-A-C	L29R	n/a	n/a	23.9	0.536	0;74	n/a	VUS (PM2)
*DNAH3*	16–20,944,699-T-C ^e^	Y4043C	7.6E−05	5.6E−04	27	0.798	0;47	n/a	VUS (PS4)
*DNAH3*	16–20,990,858-T-C ^e^	M2624V	3.3E−04	2.7E−03	23	0.798	0;55	n/a	VUS (PS4, BP4)
*DNAH8*	6–38,840,471-A-T	M2372L	3.2E−05	2.6E−04	27.1	0.675	0;45	n/a	VUS (PS4)
*HSPG2*	1–22,186,478-C-T	V1678M	8.3E−06	6.7E−05	25	0.412	1;259	n/a	Likely pathogenic (PS4, PM2)
*KCNA4*	11–30,032,402-C-G	K608N	4.7E−04	6.9E−04	23.3	0.281	0;80	VUS	VUS (PS4)
*LAMA4*	6–112,537,568-C-A	Splicing	1.2E−04	9.5E−04	31	0.73	0;61	n/a	Likely pathogenic (PS4, PM4)
*LAMA5*	20–60902366-G-A	R1679W	8.7E−03	2.1E−03	24.3	0.311	0;178	Benign	VUS (PS4, BP4)
*LAMB2*	3–49,159,606-G-A	R1591W	6.4E−05	4.6E−04	27.8	0.655	0;118	n/a	VUS (PS4)
*LTBP4*	19–41,116,501-G-T	G613V	2.4E−05	1.8E−04	29.8	0.327	0;155	VUS	VUS (PS4, PP3)
*PLEKHA7*	11–17,035,539-A-G	I73T	3.5E−05	2.2E−04	25.7	0.531	0;75	n/a	VUS (PS4)
*PRKD1*	14–30,066,918-G-T	S738Y	n/a	n/a	29.7	0.622	0;53	n/a	Likely pathogenic (PS4, PM2, PP3)
*RYR2*	1–237,777,682-G-A	G1750S	2.4E−05	3.3E−05	25.3	0.25	0;152	n/a	Likely pathogenic (PS4, PM2, PP3)
*SGCA*	17–48,252,725-A-G	N364S	2.4E−05	6.6E−05	23.8	0.789	0;84	VUS	Likely pathogenic (PS4, PM2, PP3)
*SLIT3*	5–168,093,556-C-T	S1499N	1.6E−03	4.6E−03	25.2	0.318	2;48	n/a	VUS (PP3)
*SVIL*	10–29813438-T-A	Y850F	1.4E−04	9.8E−04	21	0.358	0;62	n/a	VUS (PS4)
*SYNJ2*	6–158,464,362-C-G	D242E	3.2E−05	2.6E−04	23	0.691	0;30	n/a	VUS (PS4)
*TTN*	2–179,429,320-A-G ^e^	I18307T	6.7E−04	1.8E−03	22.9	0.354	0;80	VUS, benign, likely benign	VUS (PS4)
*TTN*	2–179,435,825-G-A ^e^	R16139W	8.8E−04	6.2E−03	22.7	0.354	0;48	VUS, benign, likely benign	VUS ()
*VDAC1*	5–133,326,592-G-A	R93C	9.3E−04	8.7E−03	26.7	0.306	0;39	n/a	VUS (BP4)

RHVs in all 693 genes in GS1 are shown in supplementary table S2

^a^Minor allele frequency (MAF) among all 125,748 exomes in GnomAD

^b^MAF among 15,308 South Asian exomes in GnomAD

^c^Loss-of-function observed/expected upper bound fraction (LOEUF)

^d^Allelic depth (AD, number of sequencing reads covering the variant) of variant in reference genome (REF) and alternative variant (ALT)

^e^These variants were identified in different patients

^f^Interpretation of variants in ClinVar (https://www.ncbi.nlm.nih.gov/clinvar/). Note: these variants might have been interpreted in a monogenic setting

^g^Interpretation of variants according to the recommendation from American College of Medical Genetics and Genomics (Richards et al. 2015)

Previous research suggest that cilia genes are enriched for RGs, suggesting that cilia genes may serve as a reservoir of rare recessive variants that can cause CHD in homozygous or compound heterozygous form (Watkins et al. 2019). This observation prompted us to investigate our gene-sets for ciliary gene enrichment. We calculated the overlap between CiliaCarta, a compendium of 935 unique cilia genes and the 693 genes in GS1. Our analysis showed significant enrichment of cilia genes in GS1 (RF = 1.6, P < 5.4e−04, 52 cilia genes).

**Fig. 3:**
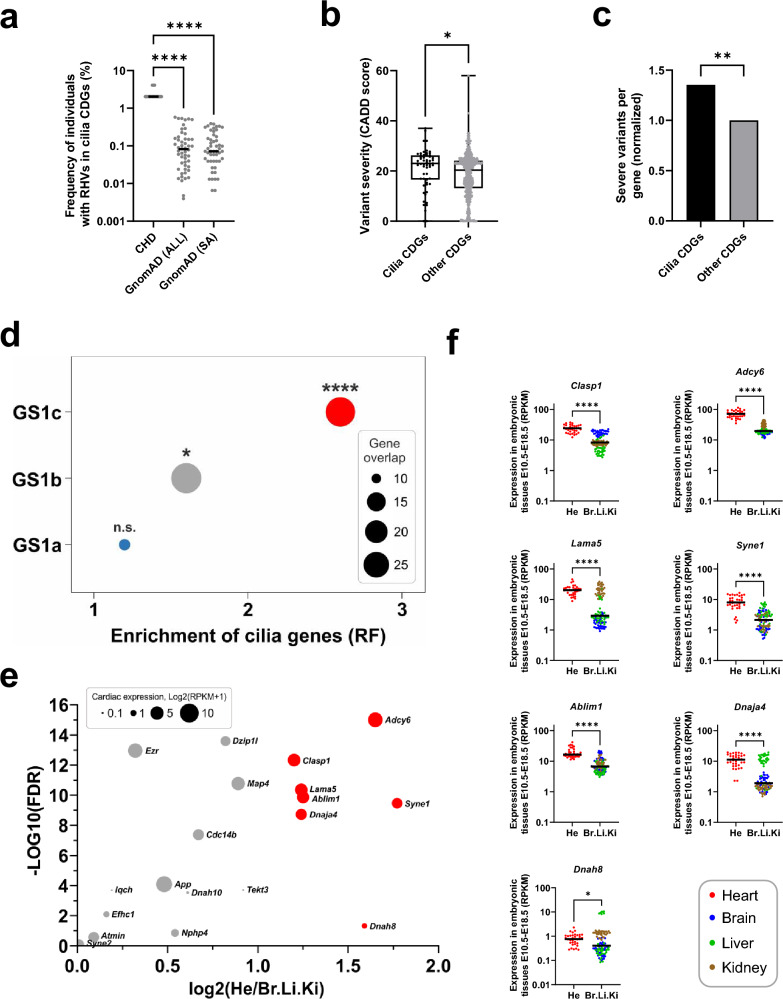
Cilia genes are enriched for rare homozygous variants. **a** Frequency of individuals with RHVs in 52 cilia CDGs. Frequency per gene in patient cohort (N = 49) was compared with frequency in GnomAD populations. GnomAD (All): Total GnomAD sample (N = 125,748). GnomAD (SA): South Asian GnomAD sub-sample (N = 15,308). Statistic comparison was performed using ANOVA (Kruskal–Wallis test). **b** Comparison of variant severity of RHVs identified in cilia CDGs (N = 52) and other CDGs (N = 641). Variant severity is indicated as CADD score. Difference between medians were determined using ANOVA (Kruskal–Wallis test). **c** Normalized comparison of severe RHVs per gene, between cilia CDGs and other CDGs. Severe variants were defined as RHVs with CADD score ≥ 21. Statistical significance of the difference was determined using Fisher’s exact test. **d** Enrichment of cilia genes among three subsets of GS1. Enrichment was calculated by comparing gene-overlap between CiliaCarta genes (N = 935) and genes within each GS1 subset. Enrichment is shown as representation factor (RF). Hypergeometric statistics was used to test the significance of the overlaps. **e** Volcano plot of 52 cilia genes showing the differene in gene expression between developing heart (He) and developing brain (Br), Liver (Li) and Kidney (Ki) in mice at E10.5–E18.5 (Cardoso-Moreira et al. 2019). X axis shows the log2 difference between average expression in He and average expression in Br, Li and Ki (only positive values shown). Y axis shows the significance, calculated as –Log10 to the false discovery rate (FDR) (Mann–Whitney U test, adjusted for multiple testing). Significant genes, with log2 difference > 1 is shown with red color. The size of the circle indicate log2 of the average expression of the gene in developing hearts. **f** Tissue comparative expression of individual genes with fold change > 1. Asterisks indicate P values: *P < 0.05, **P < 0.01, ****P < 0.0001. ns: not significant

We proceeded to compare the frequency of individuals with RHVs in each of the 52 cilia candidate genes between our patient cohort and controls from the GnomAD database (Fig. 3a) and observed a ten-fold higher frequency of individuals with RHVs in cilia genes, among our patients, compared to the control populations. Additionally, we compared the CADD scores of RHVs in the 52 cilia genes, with the 641 non-cilia genes in GS1 and observed significantly higher median CADD score for variants in the cilia genes (Fig. 3b). Furthermore, we observed enrichment of damaging variants (CADD score ≥ 21) in cilia genes (Fig. 3c).

We calculated the enrichment of cilia genes in all three GS1 gene-subsets. We did not observe significant enrichment in the GS1a gene-subset, but both GS1b and GS1c were enriched for cilia genes, with RF of 1.6 and 2.6, respectively (Fig. 3d) and we observed the strongest enrichment for cilia genes in the final subset of 23 CDGs (RF = 5.6, p < 5.2e−04). When we compared the frequency of RHVs in each of 23 cilia genes in GS1c, we observed a very high frequency in our patients compared to GnomAD controls (Fig. S5). Overall, these analyses support our hypothesis that RHVs in cilia genes are associated with CHD in our patient cohort.

To identify cilia candidate genes with heart-specific expression, we compared gene expression in developing heart with gene expression in developing brain, liver and kidney cilia genes (Fig. 3e, f). We identified seven cilia genes of which the mouse orthologue had more than two-fold higher expression in the developing heart compared to brain, liver and kidney. Of these, five are within the list of 23 CDGs (Table 2); *ABLIM1, ADCY6, CLASP1, DNAH8, LAMA5*.

Recently, we have identified *ADCY2* and *ADCY5* as CHD disease genes (Izarzugaza et al. 2020), thus *ADCY6* appeared to be an interesting disease candidate. In addition, *Adcy6* exhibited the most significant difference in expression between heart and other tissues and the highest expression in the embryonic heart among the ciliary candidate disease genes (Fig. 3e, f). However, *ADCY6* has not previously been associated with CHD or heart development. Therefore, we used cell models and zebrafish to investigate a cilia-related function of ADCY6 in heart development.

P19CL6 are teratocarcinoma derived pluripotent stem cells, which will spontaneously differentiate into beating cardiomyocytes within 12–14 days after addition of DMSO to the growth media (Clement et al. 2009a, 2013; Habara-Ohkubo 1996; Koefoed et al. 2018). During the differentiation of P19CL6 cells, a decreased expression of the stem cell marker SOX2 is concomitant with increased expression of the cardiomyocyte transcription factor GATA4 (Fig. S6a). Around day 12, development of sarcomeric structures can be evidenced by a striated pattern of Troponin I (alongside α-actinin) and spontaneously beating cardiomyocytes start to appear in the culture (Fig. S6b).

**Fig. 4:**
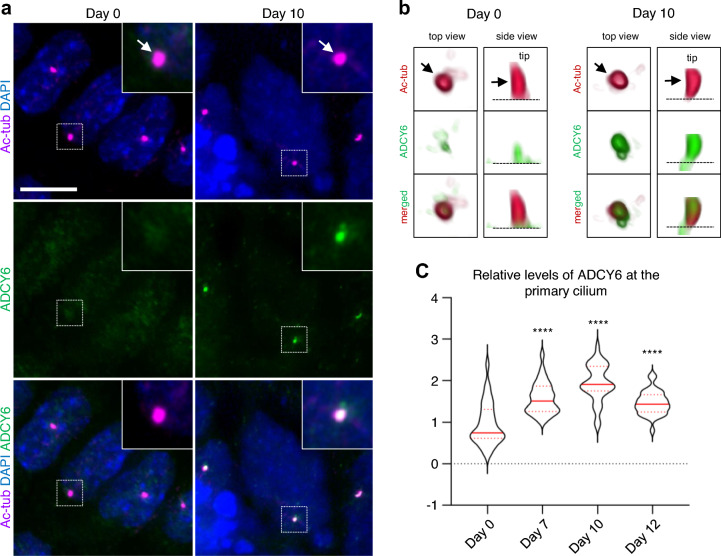
Temporal localization of ADCY6 to primary cilia during cardiomyogenesis. ADCY6 accumulate at primary cilia during differentiation of P19.CL6 cells into cardiomyocytes. **a** Representative images P19.CL6 cells at day 0 (stem cells) and day 10 (cardiomyocytes). Arrows point to primary cilia. Scale bar, 10 µm. **b** 3D visualization of representative cilia at day 0 and day 10 of differentiation. Arrow points to the primary cilium. Asterisk marks the ciliary base. **c** Violin plots of quantification of ADCY6 fluorescence levels at primary cilia during days 0, 7, 10 and 12 of differentiation. Asterisks indicate P values: ****P < 0.0001

To investigate whether ADCY6 has a potential ciliary function during cardiomyogenesis, we utilized immunofluorescence microscopy to examine ciliary localization of ADCY6 at four time points during differentiation of P19CL6 cells into cardiomyocytes. At day 0, when DMSO is added, ADCY6 was mainly localized to the base of the primary cilium in the stem cells (Fig. 4a, b). However, at two later stages during the differentiation process (day 7, 10), we observed a gradual two-fold increase in the levels of ADCY6 protein at the primary cilium, with ADCY6 localized along the length of the cilium (Fig. 4a–c). At day 12, the amount of ADCY6 was decreased to a level similar to day 7. These data demonstrate that ADCY6 localize to the primary cilium in a temporal manner during cardiomyogenesis.

**Fig. 5:**
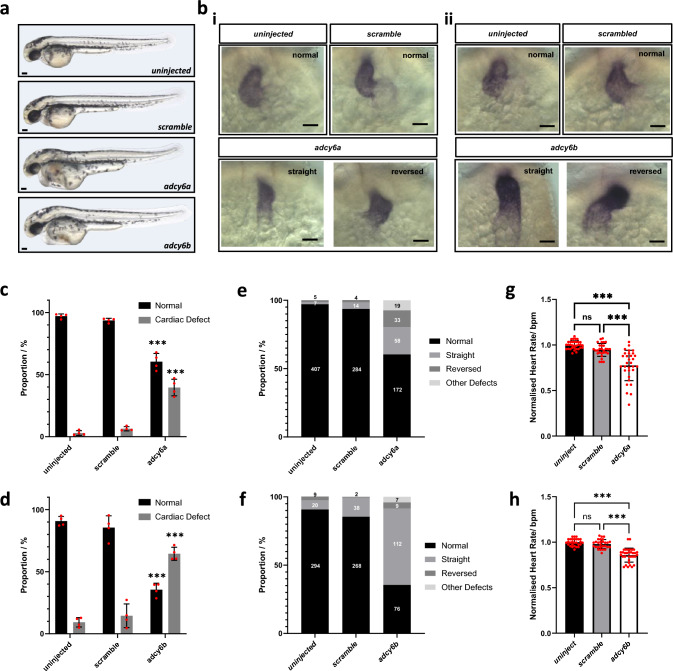
Knock-out of ADCY6 cause heart defects in zebrafish. **a** Bright-field images showing the morphology of 2 dpf uninjected, *scramble*, *adcy6a* and *adcy6b* F0 crispant zebrafish. Scale bars, 0.5 mm. **b** mRNA expression analysis of *myl7* in 2dpf crispant hearts. Upper panels of (i) and (ii) imaged from control larvae. Lower panels imaged from (i) *adcy6a* and (ii) *adcy6b* crispants. Scale bars, 50 µm. **c**, **d** Quantification of cardiac defects observed in 2 dpf **c** *adcy6a* and **d** *adcy6b* crispants upon mRNA expression analysis of *myl7*. **e**–**f** Proportion of heart phenotypes observed in **e** *adcy6a* and **f** *adcy6b* crispants. Numbers central within bars indicate number of larvae in each classification. **g**–**h** Normalized heart rate measurements in beats per minute (bpm) analyzed at 2 dpf in **g** *adcy6a* and **h** *adcy6b* crispants. Two-way ANOVA (**c**, **d**), ordinary one-way ANOVA (**g**) and Kruskal–Wallis test (**h**) used for statistical analysis. Asterisk indicate P values: ***p < 0.001. n.s.: not significant

To further study the function of *ADCY6* in heart development in vivo, we generated F0 mutant (crispant) models of the *ADCY6* zebrafish orthologues *adcy6a* and *adcy6b* (Fig. S7a, b). Crispants for *adcy6a* and *adcy6b* displayed cardiac edema, which was not observed in control samples, at 2 dpf (Fig. 5a), suggesting cardiac defects in both *adcy6* crispants.

To further delineate the cardiac phenotype of *adcy6a* and *adcy6b* crispants, we performed in situ hybridization (ISH) using a cardiac-specific *myl7* probe to label the myocardium and examined heart morphology. At 2 dpf, significant proportions of both *adcy6a* and *adcy6b* crispants displayed cardiac defects, compared to uninjected and *scramble* controls (Fig. 5b–f). A total of 39% and 63% of *adcy6a* and *adcy6b* crispants, respectively, presented with abnormal cardiac morphology, while only a small percentage of uninjected and *scramble* embryos displayed cardiac defects (Fig. 5b–f). To assess cardiac functionality, we measured the heart rate of crispant embryos at 2 dpf. Both *adcy6a* and *adcy6b* crispants presented with significantly lower heart rates when compared to controls (Fig. 5g, h).

Finally, we co-injected guides targeting both *adcy6a* and *adcy6b* simultaneously and obtained similar results as for the individual genes (Fig. S8).

In summary, CRISPR-cas9 mediated functional analysis of *ADCY6* orthologues in zebrafish models support that ADCY6 have important functions in heart development.

## Discussion

We studied a cohort of Pakistani CHD patients originating from consanguineous unions. WES analysis revealed that, on average, HBD regions covered more than 5% of the patient’s genome, thus confirming the expected consanguinity. Length of HBD segments and number of damaging RHVs per sample are comparable to results reported in a recent WES analysis of Turkish CHD patients from consanguineous families (Dong et al. 2022). The majority of heart malformations in our patient cohort, however, were simple (septal defects) as compared to a majority of complex defects, including a large number of rare laterality defects, identified in the Turkish cohort (Dong et al. 2022).

Our approach for filtering variants was centered around the idea that a substantial portion of the genetic influence on CHD in our patient population is attributable to homozygosity for recessive variants that are deleterious. To this end, we filtered for RHVs and used a combination of variant classification and loss-of-function intolerance to identify a set of 192 genes (GS1c) with high likelihood of causing CHD in our patients. We used the median value of CADD scores and LOEUF values of all 758 identified RHVs to filter the candidate genes with most likelihood of causing CHD. Enrichment analysis using datasets of known CHD disease genes confirmed that our filtering strategy was meaningful.

We identified 23 genes with specific expression in developing hearts. Of these, eight are known to cause heart defects in mouse models (*CACNA1H*, *HSPG2*, *LAMA4*, *LAMA5*, *LTBP4*, *RYR2*, *SLIT3*, *TTN*) (Blake et al. 2021) while *PRKD1*has previously been implicated in CHD. Two independent reports have identified rare homozygous loss-of-function *PRKD1* variants as causative in consanguineous families with multiplex CHD cases (Massadeh et al. 2021; Shaheen et al. 2015). In addition, one case with complex CHD was found homozygous for a rare nonsense variant in *PRKD1* in a screen of 2219 families from a highly consanguineous population (Monies et al. 2019). Interestingly, WES analysis of a large cohort of CHD patients, implicated rare de novo missense mutation in *PRKD1* in CHD, thus suggesting that *PRKD1* variants can have both recessive and dominant effects on CHD (Sifrim et al. 2016). Our patient was homozygous for a S738Y missense variant within the protein kinase domain of *PRKD1*. The variant is not present in the 125,748 exomes within the GnomAD database (Karczewski et al. 2020) and has a CADD score of 29.7, thus we find it very likely that this variant is causative in our patient.

We observed enrichment of cilia genes in GS1 and enrichment of damaging RHVs in cilia genes. Importantly, we observed strong enrichment of cilia genes in the subset of genes with high likelihood of containing disease causing genes (GS1c) in contrast to the subset of genes with low likelihood of containing disease causing genes (GS1a), where no cilia gene enrichment was evident. We calculated the strongest enrichment in the list of 23 CDGs, wherein the frequency of cilia genes are 5.6 times higher than what would be expected by chance. Based on these results, we conclude that a significant part of disease causing genes in our patients are related to cilia structure or function. Our data thus support that cilia genes are enriched for recessive genotypes in CHD (Watkins et al. 2019) and the high frequency of simple heart malformations in our cohort, and the fact that most of the genes listed in CiliaCarta are associated with structure or function of non-motile, sensory cilia, supports that the involvement of cilia genes in CHD reaches beyond laterality defects, possibly due to defects in the coordination of cellular signalling by the primary cilium during development of the myocardium (Clement et al. 2013, 2009a; Koefoed et al. 2018). Genes encoding important components of motile cilia are known to cause PCD, a rare condition characterized by laterality defects, neonatal respiratory distress, early-onset year round nasal congestion and wet cough (Horani et al. 2016; Leigh et al. 2016). None of our patients was diagnosed with laterality defects or PCD, or had cardiac defects typically associated with laterality defects. However, several patients had a history of respiratory symptoms, thus we cannot rule out the possibility of PCD in some of the patients, but we anticipate that this occurrence would be very infrequent, given the rarity of PCD as a condition, and the observation of only a few of the cardinal PCD phenotypes among these patients.

We identified *ADCY6* as a cilia candidate gene, with specific and strong expression in the developing heart. IFM analysis of differentiating P19CL6 cell cultures established that ADCY6 localize to the primary cilium during cardiomyogenesis and quantification of the level of ADCY6 protein within the cilium showed that the ciliary localization of ADCY6 increases at least two-fold during cardiomyocyte differentiation. The dynamic pattern of cilia localization supports an important ciliary function of ADCY6 during cardiomyogenesis. Functional investigation of zebrafish orthologues of *ADCY6* verified that the gene plays an important role in heart development. The identified variant (S236P) affects a conserved amino acid within the transmembrane domains of ADCY6 (Fig. S9). However, direct functional experiments are needed to confirm that this variant affects the function of ADCY6.

*ADCY6* encodes an adenylyl cyclase, which have been shown to localize to the primary cilium in neuronal precursors of the developing cerebellum and neural tube, where it appears to function as an inhibitor of hedgehog (Hh) signaling (Vuolo et al. 2015). Hh signaling plays major roles in heart development and mutations of Hh signaling components have been shown to cause CHD in mouse models (Briggs et al. 2016; Li et al. 2015; Washington Smoak et al. 2005). Furthermore, recent research suggest that Hh signaling regulates the timing of differentiation of second heart field (SHF) cardiomyocyte progenitors (Rowton et al. 2022). In this context, the dynamic localization pattern of ADCY6 at the primary cilium during cardiomyocyte differentiation of P19CL6 cells might indicate that ADCY6 is involved in regulation of Hh signaling during cardiomyogenesis. However, this hypothesis needs to be addressed by further experiments using appropriate model systems for analysis of SHF progenitor differentiation.

## Strengths and weaknesses

A strength to our work is that the genetic analysis is based on a unique sample of patients from consanguineous families. Our analysis is based on the hypothesis that recessive inherited variants add significantly to the cause of heart malformations in patients from consanguineous families. Such recessive variants are expected to occur as homozygous genotypes in affected individuals, within HBD regions, which make up only a small fraction of an individual´s genome (5–6%). This reduced the number of candidate disease genes significantly, making it easier to identify potential causes. In addition, we applied a novel approach for reducing the number of candidate genes, by combining CADD score for variants with LOEUF score for genes, followed by filtering for candidate genes with specific expression in the developing heart. However, it is important to recognize that monogenic causes of CHD are extremely rare, and despite the confirmed consanguinity of the analyzed families, we do not anticipate the CHD in these patients to have a monogenic origin. Consequently, establishing the causality of individual variants listed in Tables 2 and S2 is not feasible.

The occurrence of several siblings with CHD in a consanguineous family would have supported our hypothesis and strengthened variant causality, but we did only observe one affected individual in the majority of families. This could be due to various reasons, such as a possible oligogenic architecture of CHD (Pierpont et al. 2018), incomplete information regarding spontaneous abortions in the families and bias in the selection of patients, which were recruited from hospitals in major cities. Availability of WES data from the parents could have strengthened the genetic analysis by confirming HDB of variants and eliminating the possibility that some of our ROH might be regions with heterozygous genomic deletion. Pathogenic rare copy number variants (CNVs) are present in 3–10% of patients with isolated CHD (Pierpont et al. 2018), thus de novo CNVs or CNVs with reduced penetrance in the healthy parents could in principle be causative in some of our patients. We also acknowledge the lack of an ethically matched control cohort and the use of GnomAD South Asian population as a substitute, which may not be completely representative of the Pakistani population and may not account for compound heterozygous genotypes. And finally, a formal evaluation for PCD was not performed at the time of sampling, thus we cannot exclude that one or more of the patients may have suffered from PCD. These limitations should be taken into consideration when interpreting our findings.

## Future directions

Our results suggest several novel CHD candidate genes. These candidate genes need further validation in independent cohorts or by functional studies in cell or animal models.

Our data confirm a significant role of the primary cilium as a signaling hub in heart development and CHD. Unravelling of the mechanisms of ciliary coordination of cell signaling in heart development remain a major challenge for understanding heart development and the pathomechanisms involved in CHD and experiments with mouse models is warranted to confirm the association between cilia related genes and septal defects, suggested by our study. Our current knowledge about the spatio-temporal coordination of cardiac developmental networks by the primary cilium, including regulation of crosstalk between signaling pathways, is currently very limited and experimental research addressing this field is warranted. In addition, the composition of signaling components within the cilium is very dynamic and dependent on cell-type and the surrounding tissue, which adds another level of complexity (Mill et al. 2023).

In summary, our genetic analysis of a cohort of CHD probands, originating from consanguineous unions, led to the identification of a 23 candidate disease genes, of which six are known ciliary genes. Our results confirm an important role for cilia genes in CHD.

## Supplementary Information

Below is the link to the electronic supplementary material.

Supplementary file1 (PDF 6422 KB)

Supplementary file2 (XLSX 126 KB)

## Data Availability

Individual exome sequencing data cannot be shared due to concerns over patient privacy. Other data generated or analyzed during this study are included in the main paper, its additional files or available on request.

## References

[CR1] Anvarian Z, Mykytyn K, Mukhopadhyay S, Pedersen LB, Christensen ST (2019) Cellular signalling by primary cilia in development, organ function and disease. Nat Rev Nephrol 15:199–219. 10.1038/s41581-019-0116-9 30733609 10.1038/s41581-019-0116-9 PMC6426138

[CR2] Audain E, Wilsdon A, Breckpot J, Izarzugaza JMG, Fitzgerald TW, Kahlert AK, Sifrim A, Wünnemann F, Perez-Riverol Y, Abdul-Khaliq H, Bak M, Bassett AS, Benson DW, Berger F, Daehnert I, Devriendt K, Dittrich S, Daubeney PE, Garg V, Hackmann K, Hoff K, Hofmann P, Dombrowsky G, Pickardt T, Bauer U, Keavney BD, Klaassen S, Kramer HH, Marshall CR, Milewicz DM, Lemaire S, Coselli JS, Mitchell ME, Tomita-Mitchell A, Prakash SK, Stamm K, Stewart AFR, Silversides CK, Siebert R, Stiller B, Rosenfeld JA, Vater I, Postma AV, Caliebe A, Brook JD, Andelfinger G, Hurles ME, Thienpont B, Larsen LA, Hitz MP (2021) Integrative analysis of genomic variants reveals new associations of candidate haploinsufficient genes with congenital heart disease. PLoS Genet 17:e1009679. 10.1371/journal.pgen.1009679 34324492 10.1371/journal.pgen.1009679 PMC8354477

[CR3] Bittles AH, Black ML (2010) Evolution in health and medicine Sackler colloquium: consanguinity, human evolution, and complex diseases. Proc Natl Acad Sci USA 107(Suppl 1):1779–1786. 10.1073/pnas.0906079106 19805052 10.1073/pnas.0906079106 PMC2868287

[CR4] Blake JA, Baldarelli R, Kadin JA, Richardson JE, Smith CL, Bult CJ (2021) Mouse Genome Database (MGD): knowledgebase for mouse-human comparative biology. Nucleic Acids Res 49:D981-d987. 10.1093/nar/gkaa1083 33231642 10.1093/nar/gkaa1083 PMC7779030

[CR5] Briggs LE, Burns TA, Lockhart MM, Phelps AL, Van den Hoff MJ, Wessels A (2016) Wnt/β-catenin and sonic hedgehog pathways interact in the regulation of the development of the dorsal mesenchymal protrusion. Dev Dyn 245:103–113. 10.1002/dvdy.24339 26297872 10.1002/dvdy.24339 PMC4978225

[CR6] Cardoso-Moreira M, Halbert J, Valloton D, Velten B, Chen C, Shao Y, Liechti A, Ascenção K, Rummel C, Ovchinnikova S, Mazin PV, Xenarios I, Harshman K, Mort M, Cooper DN, Sandi C, Soares MJ, Ferreira PG, Afonso S, Carneiro M, Turner JMA, VandeBerg JL, Fallahshahroudi A, Jensen P, Behr R, Lisgo S, Lindsay S, Khaitovich P, Huber W, Baker J, Anders S, Zhang YE, Kaessmann H (2019) Gene expression across mammalian organ development. Nature 571:505–509. 10.1038/s41586-019-1338-5 31243369 10.1038/s41586-019-1338-5 PMC6658352

[CR7] Cingolani P, Platts A, le Wang L, Coon M, Nguyen T, Wang L, Land SJ, Lu X, Ruden DM (2012) A program for annotating and predicting the effects of single nucleotide polymorphisms, SnpEff: SNPs in the genome of *Drosophila melanogaster* strain w1118; iso-2; iso-3. Fly (Austin) 6:80–92. 10.4161/fly.19695 22728672 10.4161/fly.19695 PMC3679285

[CR8] Clement CA, Kristensen SG, Møllgård K, Pazour GJ, Yoder BK, Larsen LA, Christensen ST (2009a) The primary cilium coordinates early cardiogenesis and hedgehog signaling in cardiomyocyte differentiation. J Cell Sci 122:3070–3082. 10.1242/jcs.049676 19654211 10.1242/jcs.049676 PMC2729259

[CR9] Clement CA, Larsen LA, Christensen ST (2009b) Using nucleofection of siRNA constructs for knockdown of primary cilia in P19.CL6 cancer stem cell differentiation into cardiomyocytes. Methods Cell Biol 94:181–197. 10.1016/s0091-679x(08)94009-7 20362091 10.1016/S0091-679X(08)94009-7

[CR10] Clement CA, Ajbro KD, Koefoed K, Vestergaard ML, Veland IR, Henriques de Jesus MP, Pedersen LB, Benmerah A, Andersen CY, Larsen LA, Christensen ST (2013) TGF-β signaling is associated with endocytosis at the pocket region of the primary cilium. Cell Rep 3:1806–1814. 10.1016/j.celrep.2013.05.020 23746451 10.1016/j.celrep.2013.05.020

[CR11] Concordet JP, Haeussler M (2018) CRISPOR: intuitive guide selection for CRISPR/Cas9 genome editing experiments and screens. Nucleic Acids Res 46:W242-w245. 10.1093/nar/gky354 29762716 10.1093/nar/gky354 PMC6030908

[CR12] DePristo MA, Banks E, Poplin R, Garimella KV, Maguire JR, Hartl C, Philippakis AA, del Angel G, Rivas MA, Hanna M, McKenna A, Fennell TJ, Kernytsky AM, Sivachenko AY, Cibulskis K, Gabriel SB, Altshuler D, Daly MJ (2011) A framework for variation discovery and genotyping using next-generation DNA sequencing data. Nat Genet 43:491–498. 10.1038/ng.806 21478889 10.1038/ng.806 PMC3083463

[CR13] Djenoune L, Berg K, Brueckner M, Yuan S (2022) A change of heart: new roles for cilia in cardiac development and disease. Nat Rev Cardiol 19:211–227. 10.1038/s41569-021-00635-z 34862511 10.1038/s41569-021-00635-z PMC10161238

[CR14] Dong W, Kaymakcalan H, Jin SC, Diab NS, Tanıdır C, Yalcin ASY, Ercan-Sencicek AG, Mane S, Gunel M, Lifton RP, Bilguvar K, Brueckner M (2022) Mutation spectrum of congenital heart disease in a consanguineous Turkish population. Mol Genet Genomic Med 10:e1944. 10.1002/mgg3.1944 35481623 10.1002/mgg3.1944 PMC9184665

[CR15] Habara-Ohkubo A (1996) Differentiation of beating cardiac muscle cells from a derivative of P19 embryonal carcinoma cells. Cell Struct Funct 21:101–110. 10.1247/csf.21.101 8790939 10.1247/csf.21.101

[CR16] Horani A, Ferkol TW, Dutcher SK, Brody SL (2016) Genetics and biology of primary ciliary dyskinesia. Paediatr Respir Rev 18:18–24. 10.1016/j.prrv.2015.09.001 26476603 10.1016/j.prrv.2015.09.001 PMC4864047

[CR17] Izarzugaza JMG, Ellesøe SG, Doganli C, Ehlers NS, Dalgaard MD, Audain E, Dombrowsky G, Banasik K, Sifrim A, Wilsdon A, Thienpont B, Breckpot J, Gewillig M, Brook JD, Hitz MP, Larsen LA, Brunak S (2020) Systems genetics analysis identifies calcium-signaling defects as novel cause of congenital heart disease. Genome Med 12:76. 10.1186/s13073-020-00772-z 32859249 10.1186/s13073-020-00772-z PMC7453558

[CR18] Jin SC, Homsy J, Zaidi S, Lu Q, Morton S, DePalma SR, Zeng X, Qi H, Chang W, Sierant MC, Hung WC, Haider S, Zhang J, Knight J, Bjornson RD, Castaldi C, Tikhonoa IR, Bilguvar K, Mane SM, Sanders SJ, Mital S, Russell MW, Gaynor JW, Deanfield J, Giardini A, Porter GA Jr, Srivastava D, Lo CW, Shen Y, Watkins WS, Yandell M, Yost HJ, Tristani-Firouzi M, Newburger JW, Roberts AE, Kim R, Zhao H, Kaltman JR, Goldmuntz E, Chung WK, Seidman JG, Gelb BD, Seidman CE, Lifton RP, Brueckner M (2017) Contribution of rare inherited and de novo variants in 2,871 congenital heart disease probands. Nat Genet 49:1593–1601. 10.1038/ng.3970 28991257 10.1038/ng.3970 PMC5675000

[CR19] Karczewski KJ, Francioli LC, Tiao G, Cummings BB, Alföldi J, Wang Q, Collins RL, Laricchia KM, Ganna A, Birnbaum DP, Gauthier LD, Brand H, Solomonson M, Watts NA, Rhodes D, Singer-Berk M, England EM, Seaby EG, Kosmicki JA, Walters RK, Tashman K, Farjoun Y, Banks E, Poterba T, Wang A, Seed C, Whiffin N, Chong JX, Samocha KE, Pierce-Hoffman E, Zappala Z, O’Donnell-Luria AH, Minikel EV, Weisburd B, Lek M, Ware JS, Vittal C, Armean IM, Bergelson L, Cibulskis K, Connolly KM, Covarrubias M, Donnelly S, Ferriera S, Gabriel S, Gentry J, Gupta N, Jeandet T, Kaplan D, Llanwarne C, Munshi R, Novod S, Petrillo N, Roazen D, Ruano-Rubio V, Saltzman A, Schleicher M, Soto J, Tibbetts K, Tolonen C, Wade G, Talkowski ME, Neale BM, Daly MJ, MacArthur DG (2020) The mutational constraint spectrum quantified from variation in 141,456 humans. Nature 581:434–443. 10.1038/s41586-020-2308-7 32461654 10.1038/s41586-020-2308-7 PMC7334197

[CR20] Kimmel CB, Ballard WW, Kimmel SR, Ullmann B, Schilling TF (1995) Stages of embryonic development of the zebrafish. Dev Dyn 203:253–310. 10.1002/aja.1002030302 8589427 10.1002/aja.1002030302

[CR21] Kircher M, Witten DM, Jain P, O’Roak BJ, Cooper GM, Shendure J (2014) A general framework for estimating the relative pathogenicity of human genetic variants. Nat Genet 46:310–315. 10.1038/ng.2892 24487276 10.1038/ng.2892 PMC3992975

[CR22] Koefoed K, Veland IR, Pedersen LB, Larsen LA, Christensen ST (2014) Cilia and coordination of signaling networks during heart development. Organogenesis 10:108–125. 10.4161/org.27483 24345806 10.4161/org.27483 PMC4049888

[CR23] Koefoed K, Skat-Rørdam J, Andersen P, Warzecha CB, Pye M, Andersen TA, Ajbro KD, Bendsen E, Narimatsu M, Vilhardt F, Pedersen LB, Wrana JL, Anderson RH, Møllgård K, Christensen ST, Larsen LA (2018) The E3 ubiquitin ligase SMURF1 regulates cell-fate specification and outflow tract septation during mammalian heart development. Sci Rep 8:9542. 10.1038/s41598-018-27854-8 29934521 10.1038/s41598-018-27854-8 PMC6015040

[CR24] Kroll F, Powell GT, Ghosh M, Gestri G, Antinucci P, Hearn TJ, Tunbak H, Lim S, Dennis HW, Fernandez JM, Whitmore D, Dreosti E, Wilson SW, Hoffman EJ, Rihel J (2021) A simple and effective F0 knockout method for rapid screening of behaviour and other complex phenotypes. Elife. 10.7554/eLife.59683 33416493 10.7554/eLife.59683 PMC7793621

[CR25] Leigh MW, Ferkol TW, Davis SD, Lee HS, Rosenfeld M, Dell SD, Sagel SD, Milla C, Olivier KN, Sullivan KM, Zariwala MA, Pittman JE, Shapiro AJ, Carson JL, Krischer J, Hazucha MJ, Knowles MR (2016) Clinical features and associated likelihood of primary ciliary dyskinesia in children and adolescents. Ann Am Thorac Soc 13:1305–1313. 10.1513/AnnalsATS.201511-748OC 27070726 10.1513/AnnalsATS.201511-748OC PMC5021075

[CR26] Li H, Durbin R (2010) Fast and accurate long-read alignment with Burrows-Wheeler transform. Bioinformatics 26:589–595. 10.1093/bioinformatics/btp698 20080505 10.1093/bioinformatics/btp698 PMC2828108

[CR27] Li Y, Klena NT, Gabriel GC, Liu X, Kim AJ, Lemke K, Chen Y, Chatterjee B, Devine W, Damerla RR, Chang C, Yagi H, San Agustin JT, Thahir M, Anderton S, Lawhead C, Vescovi A, Pratt H, Morgan J, Haynes L, Smith CL, Eppig JT, Reinholdt L, Francis R, Leatherbury L, Ganapathiraju MK, Tobita K, Pazour GJ, Lo CW (2015) Global genetic analysis in mice unveils central role for cilia in congenital heart disease. Nature 521:520–524. 10.1038/nature14269 25807483 10.1038/nature14269 PMC4617540

[CR28] Li AH, Hanchard NA, Azamian M, D’Alessandro LCA, Coban-Akdemir Z, Lopez KN, Hall NJ, Dickerson H, Nicosia A, Fernbach S, Boone PM, Gambin T, Karaca E, Gu S, Yuan B, Jhangiani SN, Doddapaneni H, Hu J, Dinh H, Jayaseelan J, Muzny D, Lalani S, Towbin J, Penny D, Fraser C, Martin J, Lupski JR, Gibbs RA, Boerwinkle E, Ware SM, Belmont JW (2019) Genetic architecture of laterality defects revealed by whole exome sequencing. Eur J Hum Genet 27:563–573. 10.1038/s41431-018-0307-z 30622330 10.1038/s41431-018-0307-z PMC6460585

[CR29] Liu Y, Chen S, Zühlke L, Black GC, Choy MK, Li N, Keavney BD (2019) Global birth prevalence of congenital heart defects 1970–2017: updated systematic review and meta-analysis of 260 studies. Int J Epidemiol 48:455–463. 10.1093/ije/dyz009 30783674 10.1093/ije/dyz009 PMC6469300

[CR30] Massadeh S, Albeladi M, Albesher N, Alhabshan F, Kampe KD, Chaikhouni F, Kabbani MS, Beetz C, Alaamery M (2021) Novel autosomal recessive splice-altering variant in PRKD1 is associated with congenital heart disease. Genes (Basel). 10.3390/genes12050612 33919081 10.3390/genes12050612 PMC8143129

[CR31] McKenna A, Hanna M, Banks E, Sivachenko A, Cibulskis K, Kernytsky A, Garimella K, Altshuler D, Gabriel S, Daly M, DePristo MA (2010) The genome analysis toolkit: a MapReduce framework for analyzing next-generation DNA sequencing data. Genome Res 20:1297–1303. 10.1101/gr.107524.110 20644199 10.1101/gr.107524.110 PMC2928508

[CR32] Mill P, Christensen ST, Pedersen LB (2023) Primary cilia as dynamic and diverse signalling hubs in development and disease. Nat Rev Genet 24:421–441. 10.1038/s41576-023-00587-9 37072495 10.1038/s41576-023-00587-9 PMC7615029

[CR33] Monies D, Abouelhoda M, Assoum M, Moghrabi N, Rafiullah R, Almontashiri N, Alowain M, Alzaidan H, Alsayed M, Subhani S, Cupler E, Faden M, Alhashem A, Qari A, Chedrawi A, Aldhalaan H, Kurdi W, Khan S, Rahbeeni Z, Alotaibi M, Goljan E, Elbardisy H, ElKalioby M, Shah Z, Alruwaili H, Jaafar A, Albar R, Akilan A, Tayeb H, Tahir A, Fawzy M, Nasr M, Makki S, Alfaifi A, Akleh H, Yamani S, Bubshait D, Mahnashi M, Basha T, Alsagheir A, Abu Khaled M, Alsaleem K, Almugbel M, Badawi M, Bashiri F, Bohlega S, Sulaiman R, Tous E, Ahmed S, Algoufi T, Al-Mousa H, Alaki E, Alhumaidi S, Alghamdi H, Alghamdi M, Sahly A, Nahrir S, Al-Ahmari A, Alkuraya H, Almehaidib A, Abanemai M, Alsohaibaini F, Alsaud B, Arnaout R, Abdel-Salam GMH, Aldhekri H, AlKhater S, Alqadi K, Alsabban E, Alshareef T, Awartani K, Banjar H, Alsahan N, Abosoudah I, Alashwal A, Aldekhail W, Alhajjar S, Al-Mayouf S, Alsemari A, Alshuaibi W, Altala S, Altalhi A, Baz S, Hamad M, Abalkhail T, Alenazi B, Alkaff A, Almohareb F, Al Mutairi F, Alsaleh M, Alsonbul A, Alzelaye S, Bahzad S, Manee AB, Jarrad O, Meriki N, Albeirouti B, Alqasmi A, AlBalwi M, Makhseed N et al (2019) Lessons learned from large-scale, first-tier clinical exome sequencing in a highly consanguineous population. Am J Hum Genet 104:1182–1201. 10.1016/j.ajhg.2019.04.011 31130284 10.1016/j.ajhg.2019.04.011 PMC6562004

[CR34] Morton SU, Quiat D, Seidman JG, Seidman CE (2022) Genomic frontiers in congenital heart disease. Nat Rev Cardiol 19:26–42. 10.1038/s41569-021-00587-4 34272501 10.1038/s41569-021-00587-4 PMC9236191

[CR35] Pierpont ME, Brueckner M, Chung WK, Garg V, Lacro RV, McGuire AL, Mital S, Priest JR, Pu WT, Roberts A, Ware SM, Gelb BD, Russell MW (2018) Genetic basis for congenital heart disease: revisited: a scientific statement from the American Heart Association. Circulation 138:e653–e711. 10.1161/cir.0000000000000606 30571578 10.1161/CIR.0000000000000606 PMC6555769

[CR36] Richards S, Aziz N, Bale S, Bick D, Das S, Gastier-Foster J, Grody WW, Hegde M, Lyon E, Spector E, Voelkerding K, Rehm HL (2015) Standards and guidelines for the interpretation of sequence variants: a joint consensus recommendation of the American College of Medical Genetics and Genomics and the Association for Molecular Pathology. Genet Med 17:405–424. 10.1038/gim.2015.30 25741868 10.1038/gim.2015.30 PMC4544753

[CR37] Rowton M, Perez-Cervantes C, Hur S, Jacobs-Li J, Lu E, Deng N, Guzzetta A, Hoffmann AD, Stocker M, Steimle JD, Lazarevic S, Oubaha S, Yang XH, Kim C, Yu S, Eckart H, Koska M, Hanson E, Chan SSK, Garry DJ, Kyba M, Basu A, Ikegami K, Pott S, Moskowitz IP (2022) Hedgehog signaling activates a mammalian heterochronic gene regulatory network controlling differentiation timing across lineages. Dev Cell 57:2181-2203.e9. 10.1016/j.devcel.2022.08.009 36108627 10.1016/j.devcel.2022.08.009 PMC10506397

[CR38] Seelow D, Schuelke M (2012) HomozygosityMapper2012–bridging the gap between homozygosity mapping and deep sequencing. Nucleic Acids Res 40:W516–W520. 10.1093/nar/gks487 22669902 10.1093/nar/gks487 PMC3394249

[CR39] Shaheen R, Al Hashem A, Alghamdi MH, Seidahmad MZ, Wakil SM, Dagriri K, Keavney B, Goodship J, Alyousif S, Al-Habshan FM, Alhussein K, Almoisheer A, Ibrahim N, Alkuraya FS (2015) Positional mapping of PRKD1, NRP1 and PRDM1 as novel candidate disease genes in truncus arteriosus. J Med Genet 52:322–329. 10.1136/jmedgenet-2015-102992 25713110 10.1136/jmedgenet-2015-102992

[CR40] Shieh JT, Bittles AH, Hudgins L (2012) Consanguinity and the risk of congenital heart disease. Am J Med Genet A 158a:1236–1241. 10.1002/ajmg.a.35272 22488956 10.1002/ajmg.a.35272 PMC3331952

[CR41] Sifrim A, Hitz MP, Wilsdon A, Breckpot J, Turki SH, Thienpont B, McRae J, Fitzgerald TW, Singh T, Swaminathan GJ, Prigmore E, Rajan D, Abdul-Khaliq H, Banka S, Bauer UM, Bentham J, Berger F, Bhattacharya S, Bu’Lock F, Canham N, Colgiu IG, Cosgrove C, Cox H, Daehnert I, Daly A, Danesh J, Fryer A, Gewillig M, Hobson E, Hoff K, Homfray T, Kahlert AK, Ketley A, Kramer HH, Lachlan K, Lampe AK, Louw JJ, Manickara AK, Manase D, McCarthy KP, Metcalfe K, Moore C, Newbury-Ecob R, Omer SO, Ouwehand WH, Park SM, Parker MJ, Pickardt T, Pollard MO, Robert L, Roberts DJ, Sambrook J, Setchfield K, Stiller B, Thornborough C, Toka O, Watkins H, Williams D, Wright M, Mital S, Daubeney PE, Keavney B, Goodship J, Abu-Sulaiman RM, Klaassen S, Wright CF, Firth HV, Barrett JC, Devriendt K, FitzPatrick DR, Brook JD, Hurles ME (2016) Distinct genetic architectures for syndromic and nonsyndromic congenital heart defects identified by exome sequencing. Nat Genet 48:1060–1065. 10.1038/ng.3627 27479907 10.1038/ng.3627 PMC5988037

[CR42] Stemmer M, Thumberger T, Del Sol KM, Wittbrodt J, Mateo JL (2015) CCTop: an intuitive, flexible and reliable CRISPR/Cas9 target prediction tool. PLoS ONE 10:e0124633. 10.1371/journal.pone.0124633 25909470 10.1371/journal.pone.0124633 PMC4409221

[CR43] Thisse C, Thisse B (2008) High-resolution in situ hybridization to whole-mount zebrafish embryos. Nat Protoc 3:59–69. 10.1038/nprot.2007.514 18193022 10.1038/nprot.2007.514

[CR44] Van der Auwera GA, Carneiro MO, Hartl C, Poplin R, Del Angel G, Levy-Moonshine A, Jordan T, Shakir K, Roazen D, Thibault J, Banks E, Garimella KV, Altshuler D, Gabriel S, DePristo MA (2013) From FastQ data to high confidence variant calls: the Genome Analysis Toolkit best practices pipeline. Curr Protoc Bioinform 43:11.10.1-11.10.33. 10.1002/0471250953.bi1110s43 10.1002/0471250953.bi1110s43 PMC4243306 25431634

[CR45] van der Linde D, Konings EE, Slager MA, Witsenburg M, Helbing WA, Takkenberg JJ, Roos-Hesselink JW (2011) Birth prevalence of congenital heart disease worldwide: a systematic review and meta-analysis. J Am Coll Cardiol 58:2241–2247. 10.1016/j.jacc.2011.08.025 22078432 10.1016/j.jacc.2011.08.025

[CR46] Vuolo L, Herrera A, Torroba B, Menendez A, Pons S (2015) Ciliary adenylyl cyclases control the Hedgehog pathway. J Cell Sci 128:2928–2937. 10.1242/jcs.172635 26092933 10.1242/jcs.172635

[CR47] Wamstad JA, Alexander JM, Truty RM, Shrikumar A, Li F, Eilertson KE, Ding H, Wylie JN, Pico AR, Capra JA, Erwin G, Kattman SJ, Keller GM, Srivastava D, Levine SS, Pollard KS, Holloway AK, Boyer LA, Bruneau BG (2012) Dynamic and coordinated epigenetic regulation of developmental transitions in the cardiac lineage. Cell 151:206–220. 10.1016/j.cell.2012.07.035 22981692 10.1016/j.cell.2012.07.035 PMC3462286

[CR48] Washington Smoak I, Byrd NA, Abu-Issa R, Goddeeris MM, Anderson R, Morris J, Yamamura K, Klingensmith J, Meyers EN (2005) Sonic hedgehog is required for cardiac outflow tract and neural crest cell development. Dev Biol 283:357–372. 10.1016/j.ydbio.2005.04.029 15936751 10.1016/j.ydbio.2005.04.029

[CR49] Watkins WS, Hernandez EJ, Wesolowski S, Bisgrove BW, Sunderland RT, Lin E, Lemmon G, Demarest BL, Miller TA, Bernstein D, Brueckner M, Chung WK, Gelb BD, Goldmuntz E, Newburger JW, Seidman CE, Shen Y, Yost HJ, Yandell M, Tristani-Firouzi M (2019) De novo and recessive forms of congenital heart disease have distinct genetic and phenotypic landscapes. Nat Commun 10:4722. 10.1038/s41467-019-12582-y 31624253 10.1038/s41467-019-12582-y PMC6797711

