# Supplementary material for: Rare homozygous cilia gene variants identified in consanguineous congenital heart disease patients
Source: Hum Genet. 2024 Sep 30;143(11):1323–39. doi: 10.1007/s00439-024-02703-z (PMC11522069; doi:10.1007/s00439-024-02703-z)
Supplement: Supplementary file 1 — Supplementary file1 (PDF 6422 KB) [file 439_2024_2703_MOESM1_ESM.pdf]

Rare homozygous cilia gene variants identified in consanguineous congenital heart disease patients

Daniel A. Baird, Hira Mubeen, Canan Doganli, Jasmijn B. Miltenburg, Oskar Kaaber Thomsen, Zafar Ali, Tahir Naveed, Asif ur Rehman, Shahid Mahmood Baig, Søren Tvorup Christensen, Muhammad Farooq, Lars Allan Larsen

Corresponding authors:
Lars Allan Larsen (larsal@sund.ku.dk).
Muhammad Farooq (mfarooq@gcuf.edu.pk)

Supplementary information:

Figures S1-S9

Table S1-S6

P1

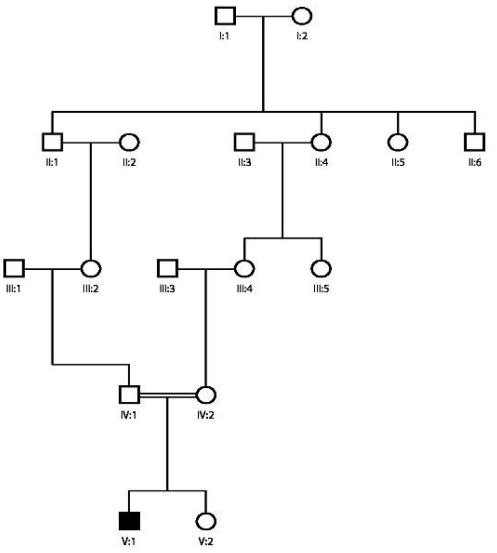

3

P2

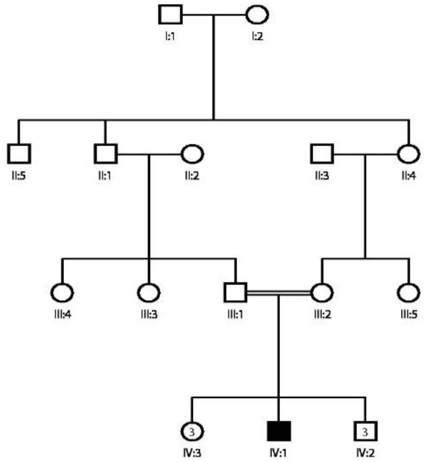

8

P3

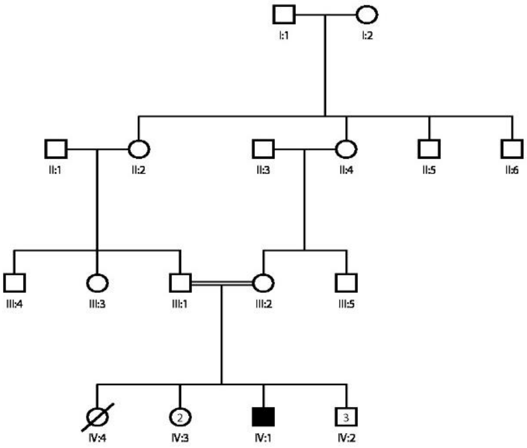

11

P4

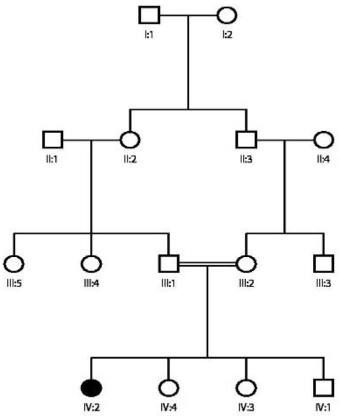

13

P5

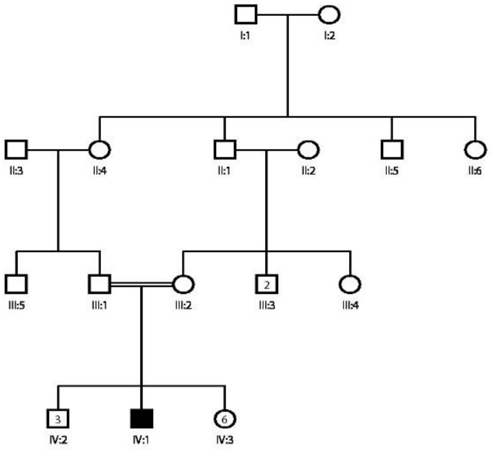

15

P6

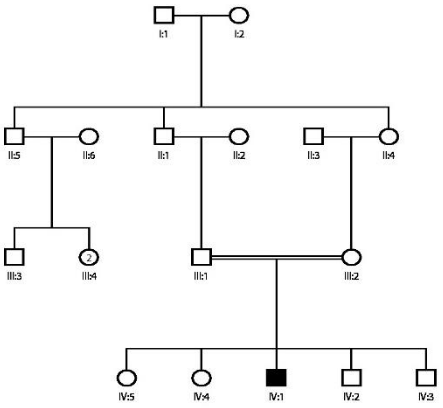

17

P7

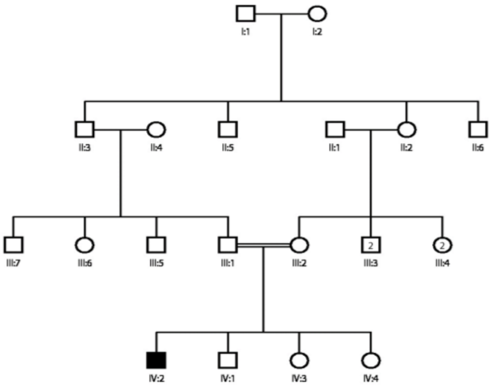

20

P8

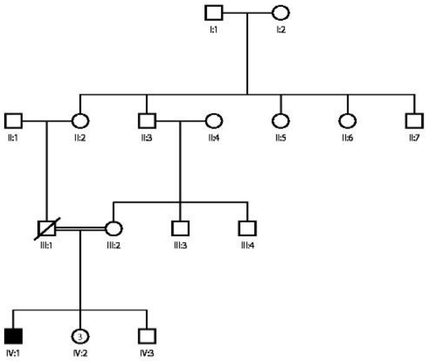

21

P9

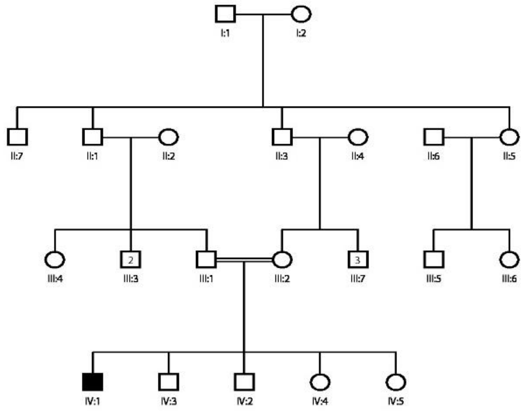

22

P10

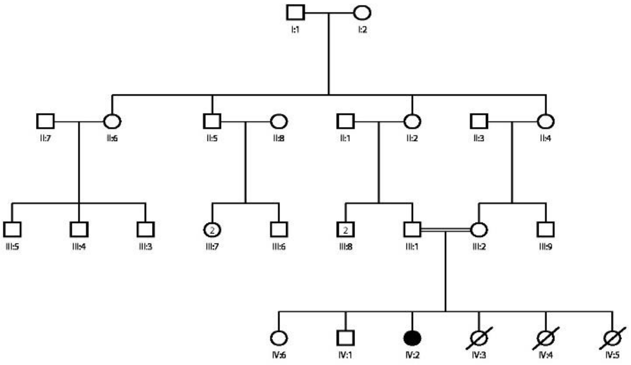

24

P11

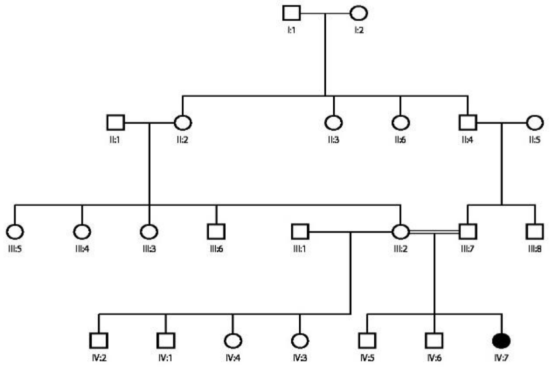

28

P12

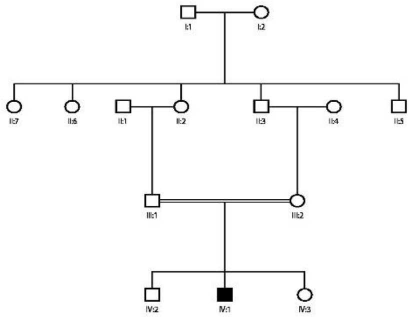

30

P13*

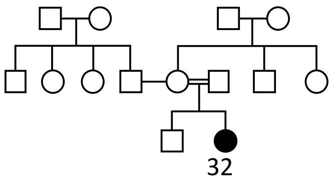

P14

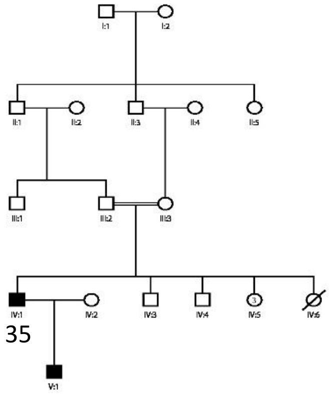

P15

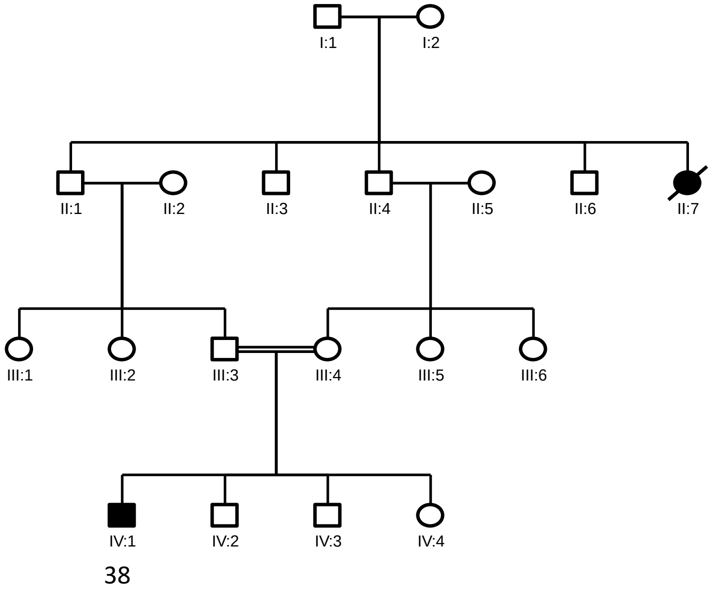

P16

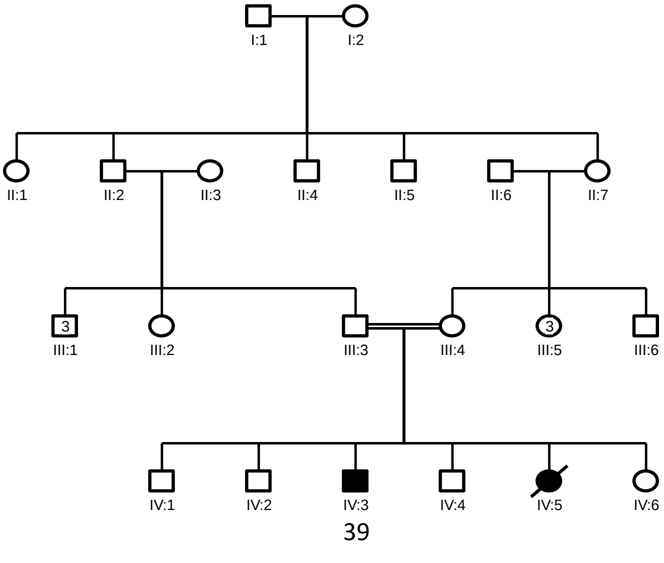

P17

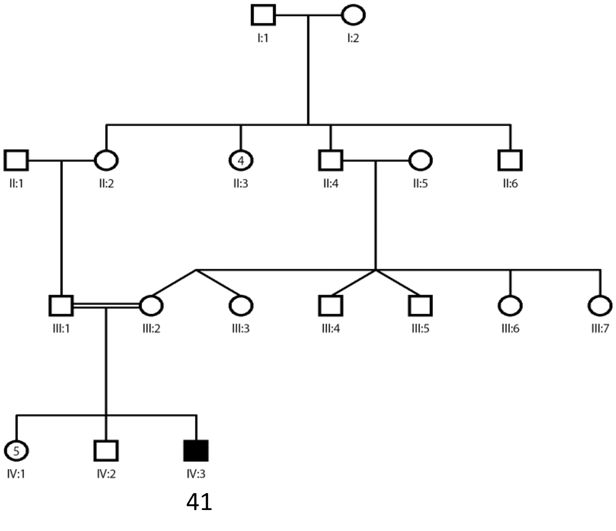

P18

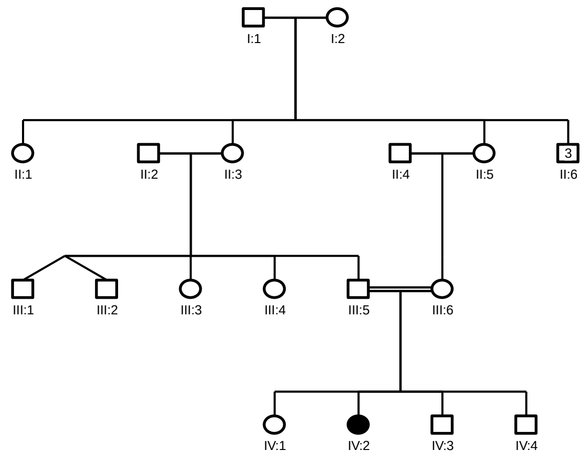

43

P19

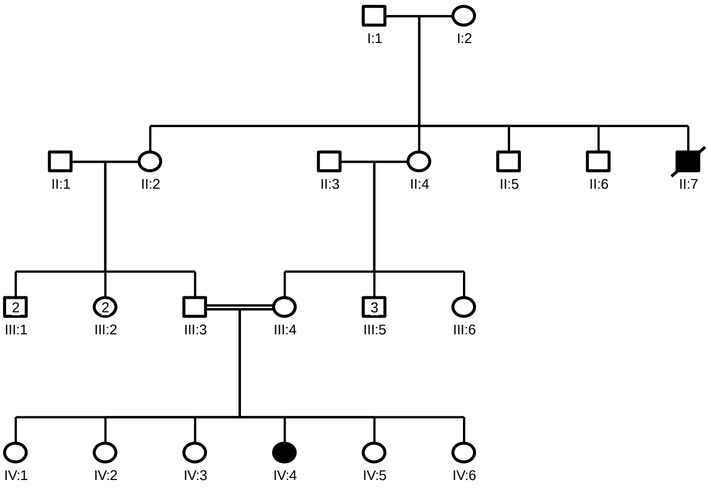

44

P20

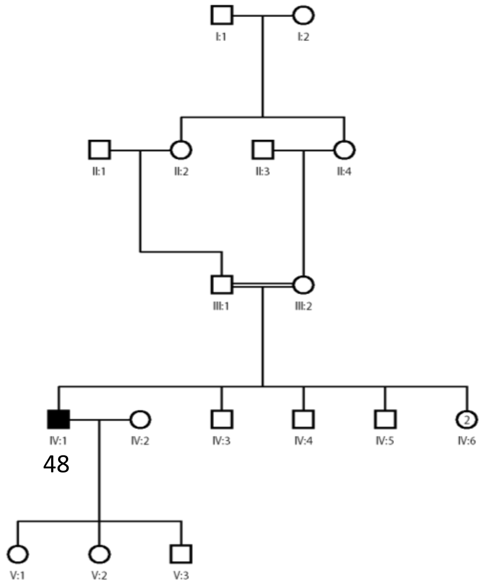

P21

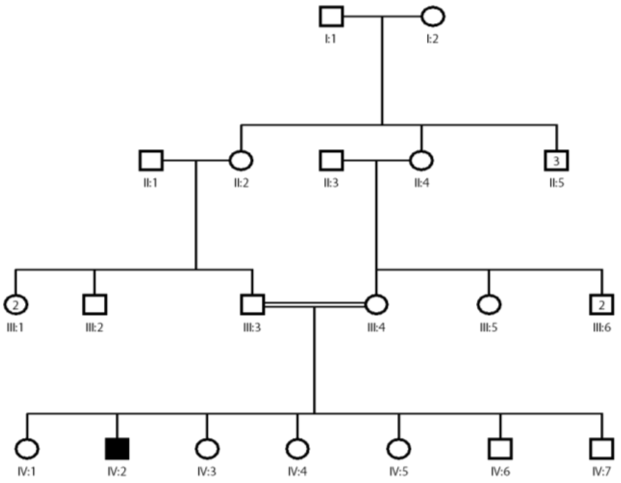

49

P22

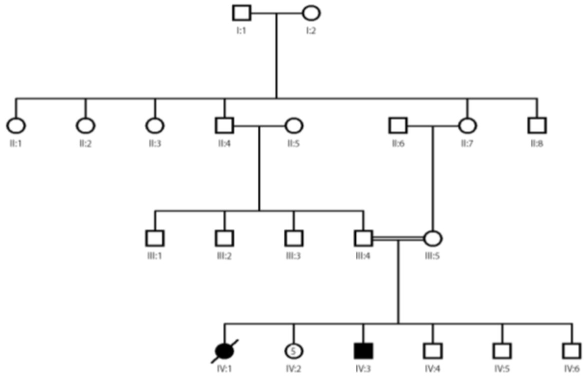

53

P23

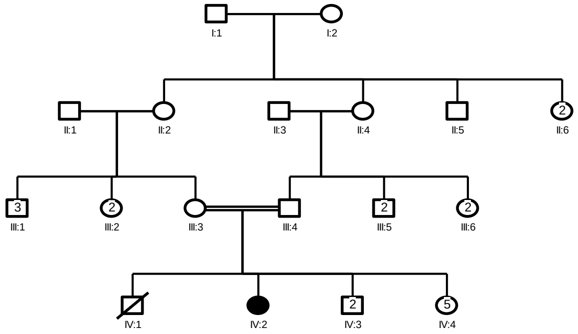

54

P24

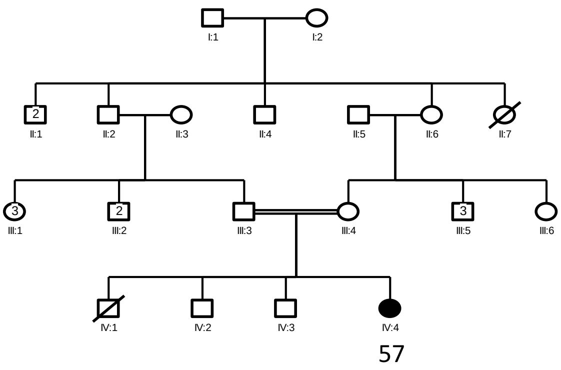

P25

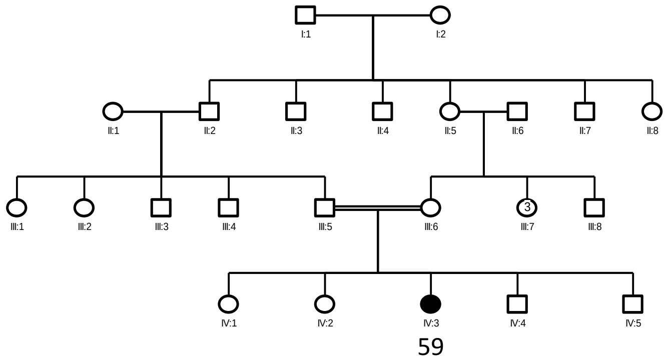

P26

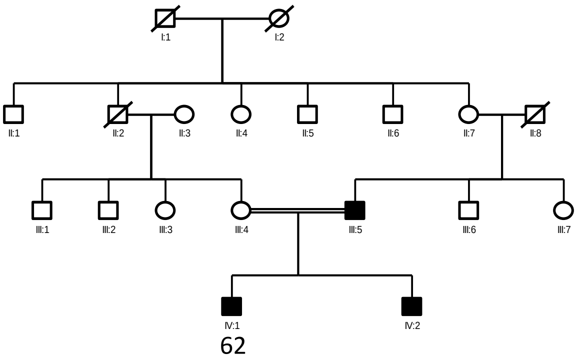

P27

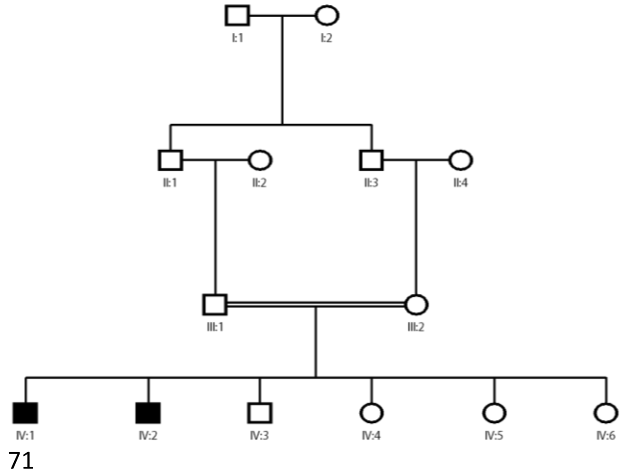

P28

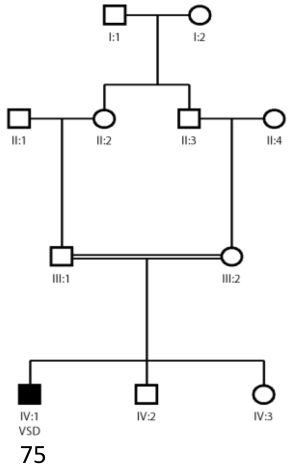

P29

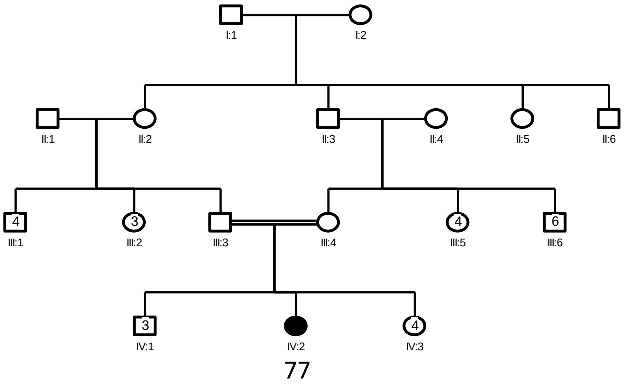

P30

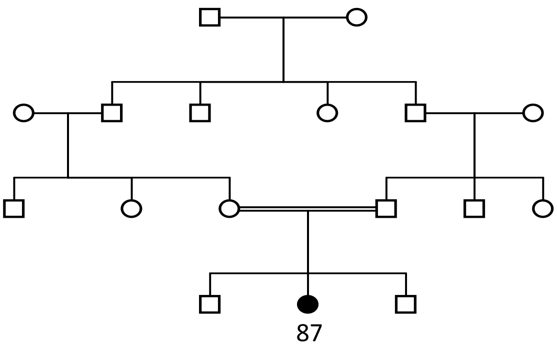

P31

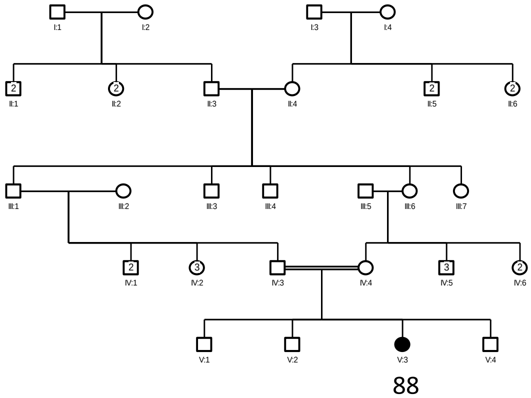

P32

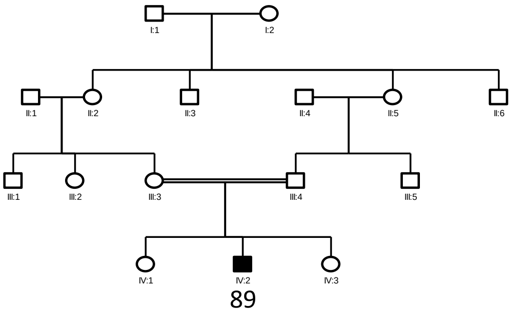

P33

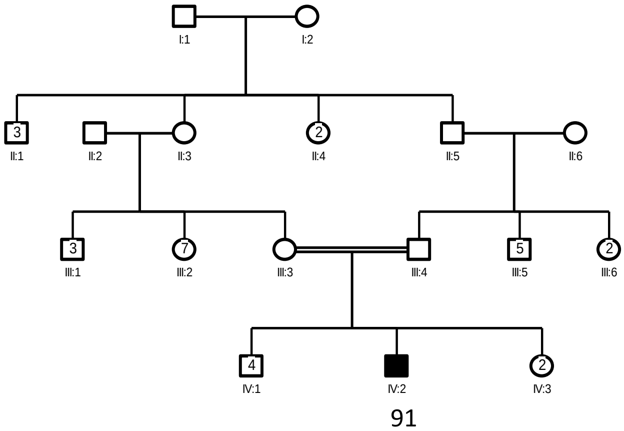

P34

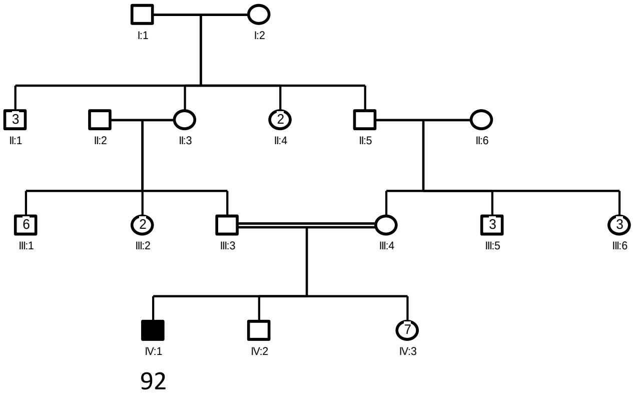

P35

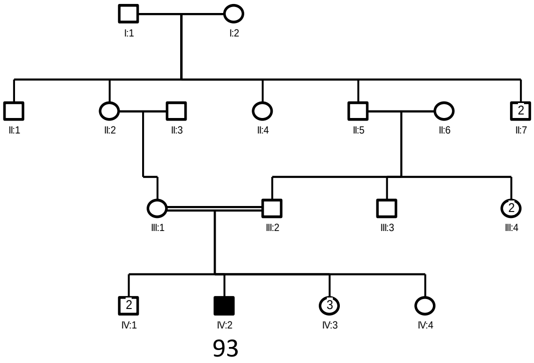

P36

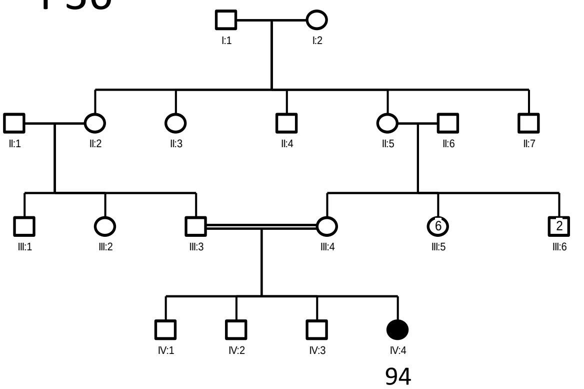

P37

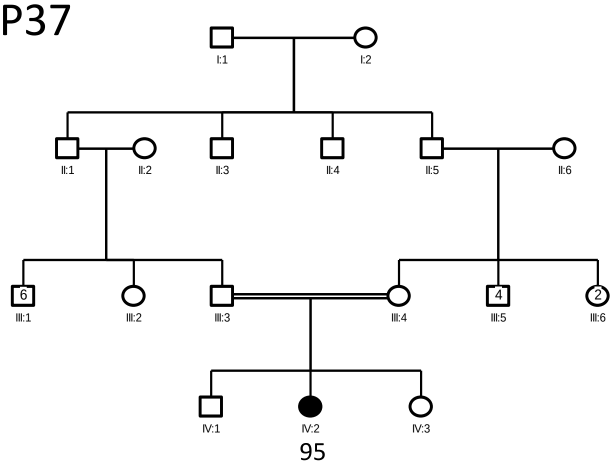

P38

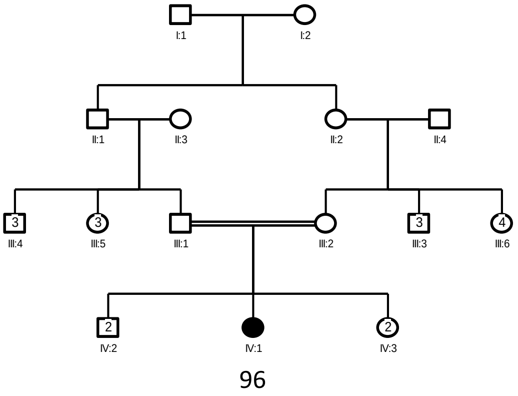

P39

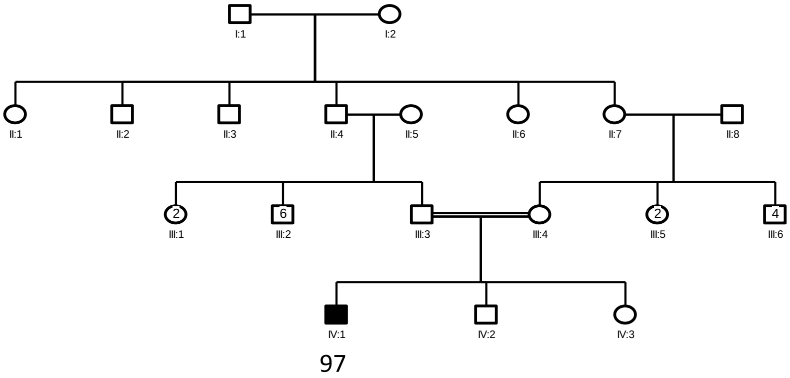

P40

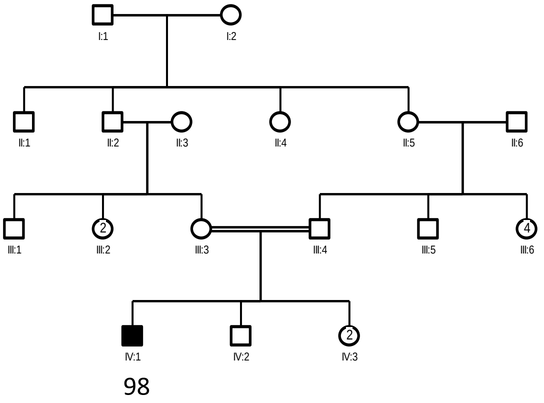

P41

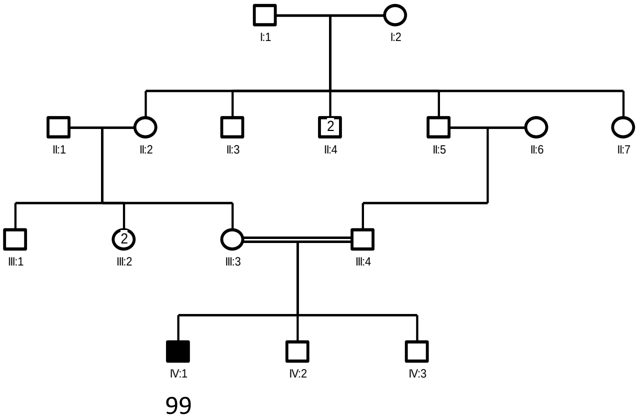

P42

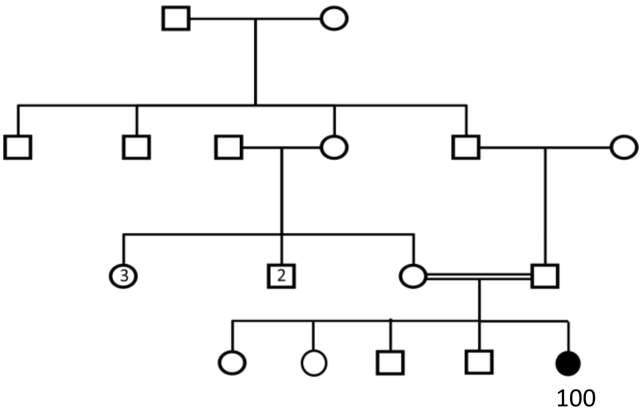

P43

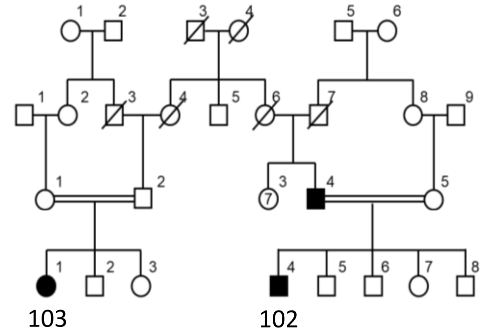

P44

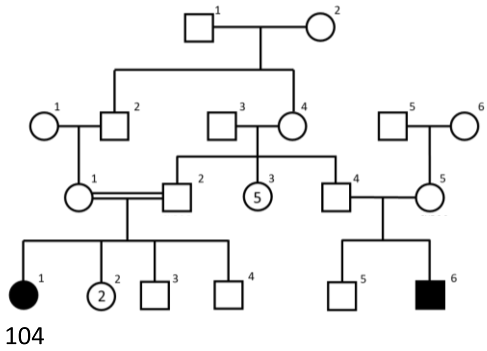

P45

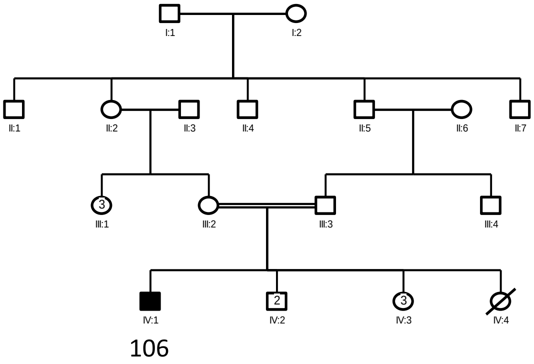

P46

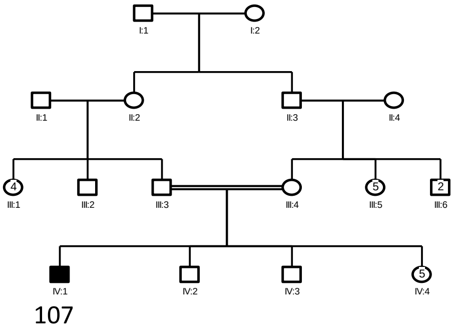

P47

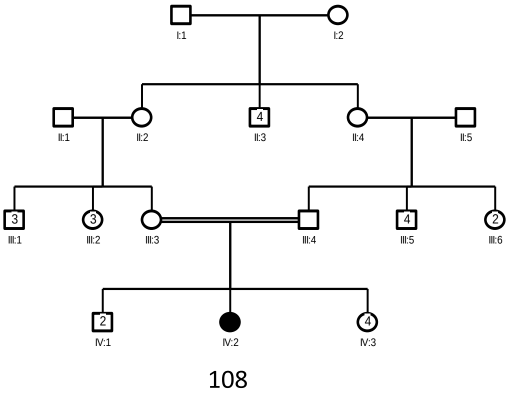

48

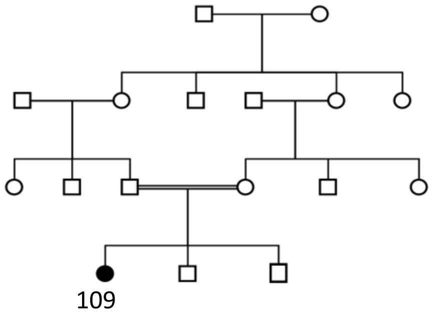

Figure S1. Pedigrees of consanguineous CHD families. Sample numbers are indicated in each family. Clinical details are listed in table S1. *: some pedigree information is missing, but consanguinity was verified by patient’s parents.

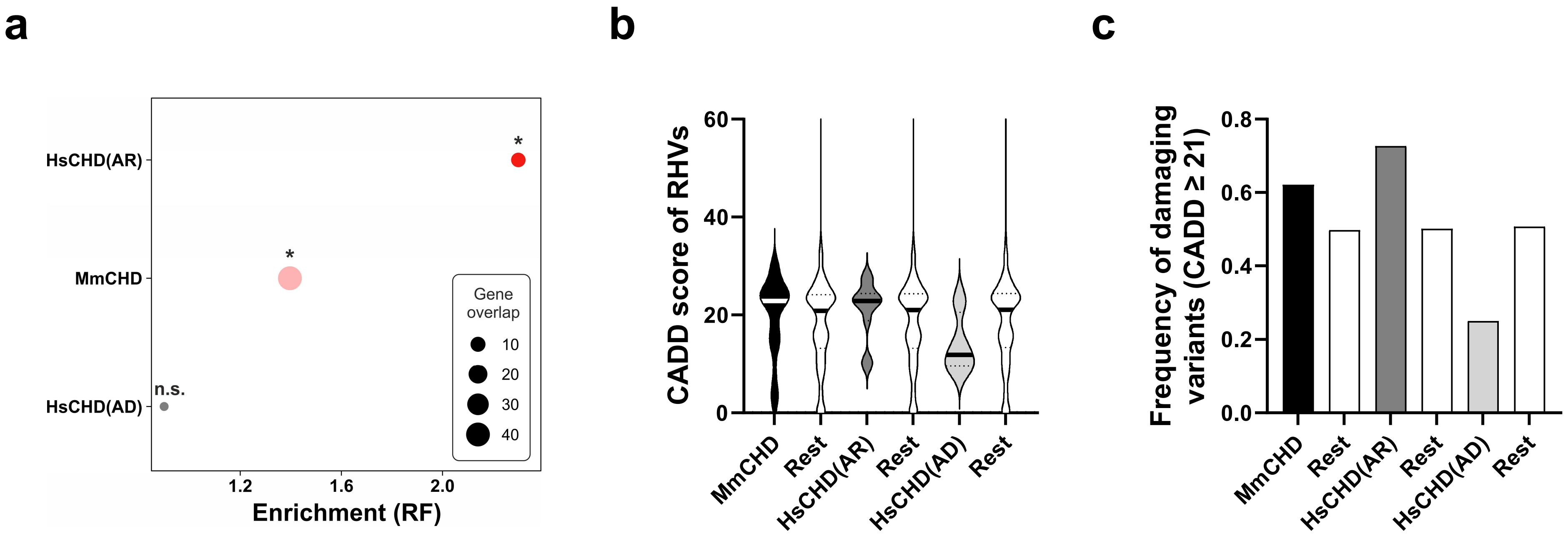

Figure S2. Enrichment of known CHD genes among 693 genes with RHVs. **a.** The overlap between lists of genes known to cause CHD in humans and mice and the list of 693 genes in GS1 (Table S1) is shown as circles. Enrichment was calculated using a hypergeometric test. Enrichment is shown as representation factor (RF). Statistical significance of the overlap is indicated. *: P<0.05, ns: not significant. HsCHD(AR) : a list of genes known to cause autosomal recessive CHD in humans (N=115), MmCHD(AR): a list of genes known to cause CHD in recessive mouse models (N=832), HsCHD(AD): a list of genes known to cause autosomal dominant CHD in humans (N=130). Genelists are shown in Table S3. **b**, **c**. Distribution of CADD scores of gene variants (**b**) and frequency of damaging variants (**c**) in overlapping genes in **a**, compared to the GS1 genes that are not overlapping (Rest). An ANOVA test determined that there is no statistical difference between medians (**b**) or frequencies (**c**).

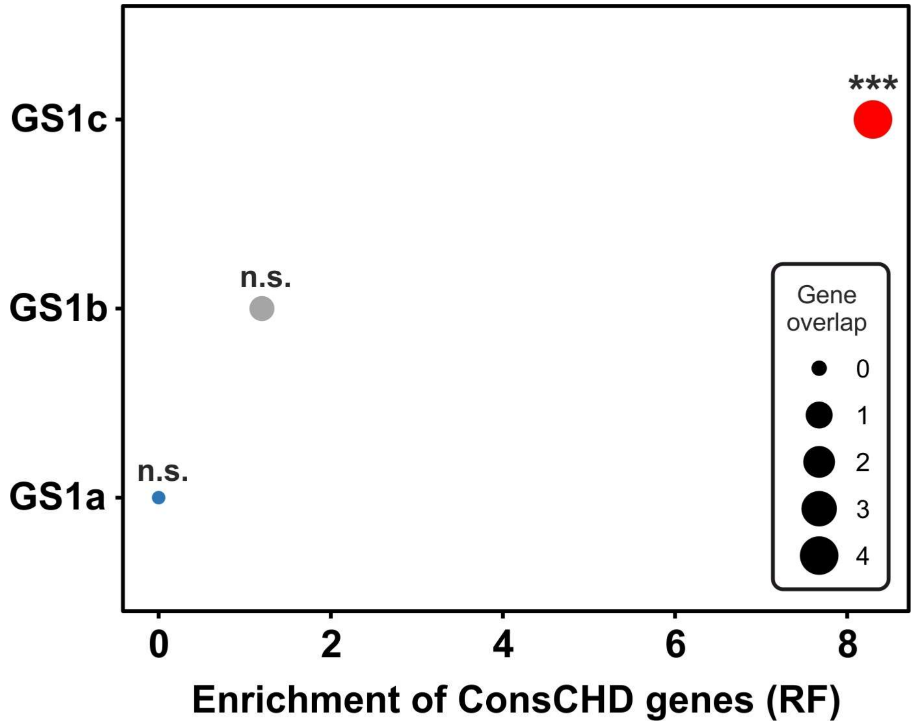

Figure S3. Enrichment of human CHD gene-set obtained from clinical exome sequencing of CHD patients from a consanguineous cohort. The set of 50 genes (ConsCHD) is listed in Supplementary Table S2. Enrichment was calculated by comparing gene-overlap between ConsCHD genes and the three sub-groups of CDGs. Enrichment is shown as representation factor (RF). A hypergeometric distribution was used to test the significance of the overlaps. Asterisks indicate P-values: *** P<0.001, n.s.: not significant.

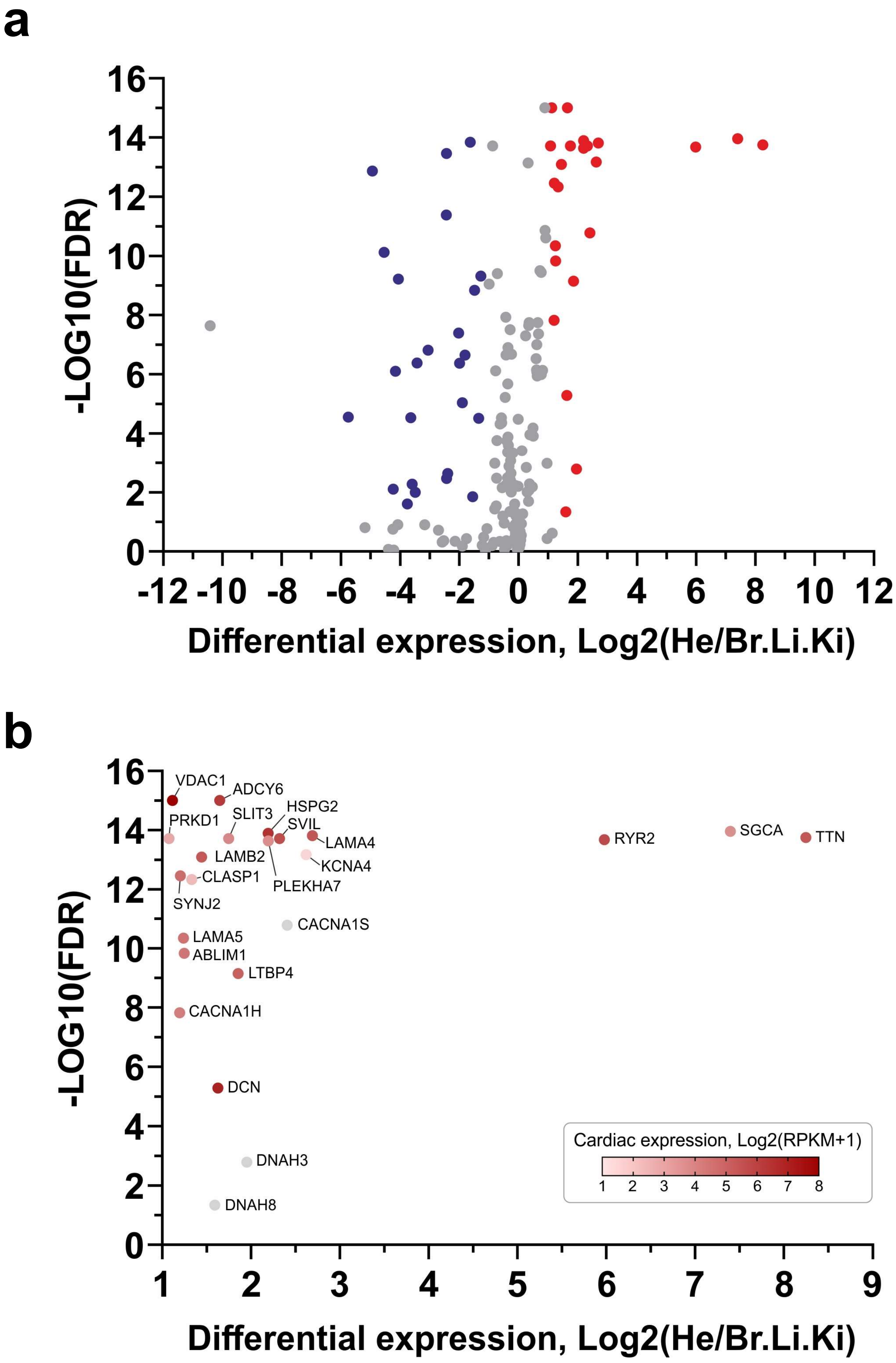

Figure S4. Cardiac specific expression of GS1c geneset. **a.** Volcano plot of genes in GS1c geneset showing the differene in gene expression between developing heart (He) and developing brain (Br), Liver (Li) and Kidney (Ki) in mice at E10.5-E18.5 . X axis shows the log2 difference between average expression in He and average expression in Br, Li and Ki. Y axis shows the significance, calculated as –Log10 to the false discovery rate (FDR) (Mann-Whitney U test, adjusted for multiple testing). Significant genes, with fold change >1 and <-1 is shown with red and blue color, respectively. **b.** Volcano plot of 23 significant genes in (**a**) with fold change >1. The color of the circle indicate Log2 of the average expression of the gene in developing hearts.

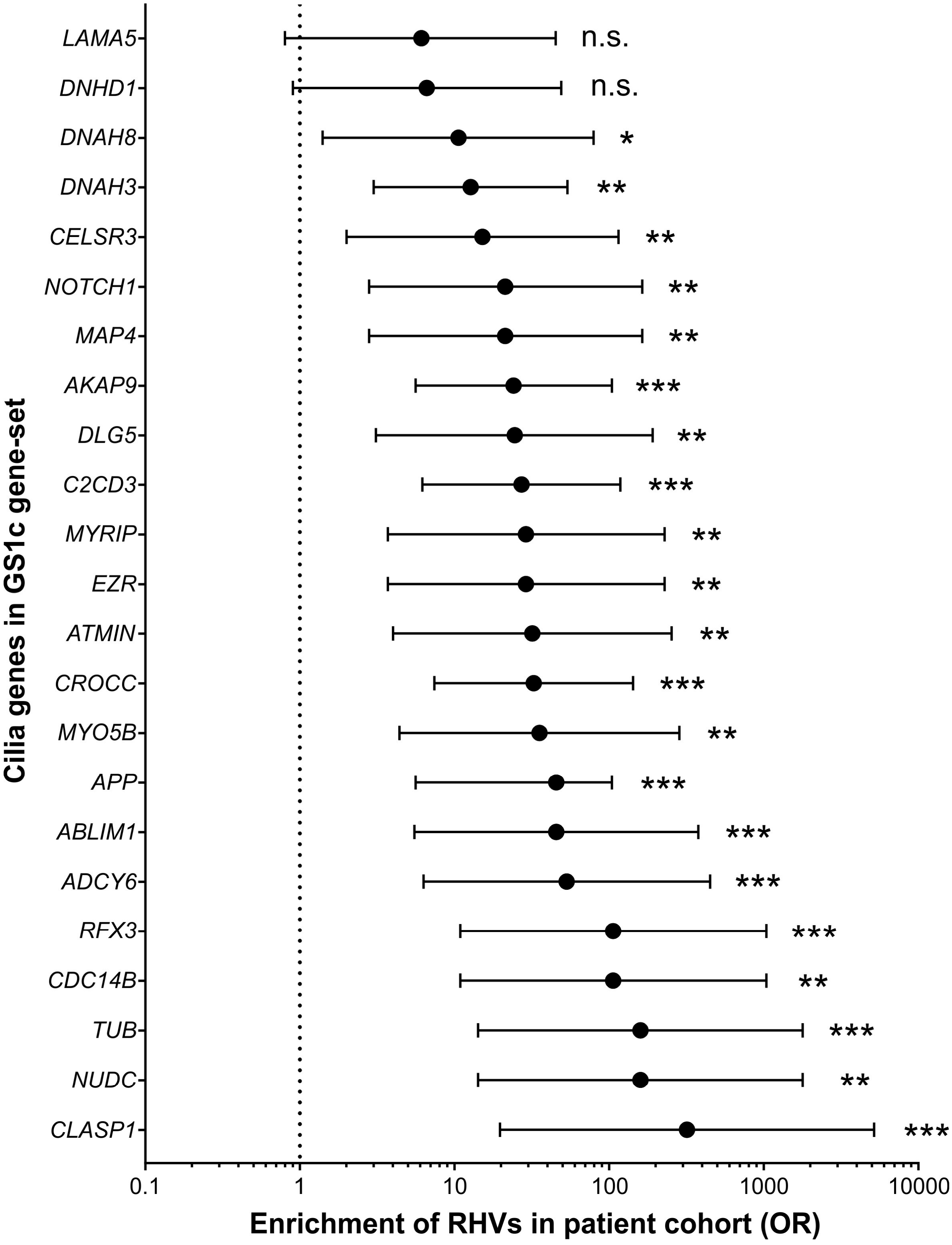

Figure S5. Forest plot of Enrichment of RHVs in each of 23 cilia genes in our patient cohort. The frequency of RHVs in patients were compared with the frequency in 15,308 South Asian controls (GnomAD v2.1.1). Enrichment was calculated as an odds ratio (circles)(medcalc.org/calc/odds_ratio). The 95% confidence interval is shown with bars. Asterisks indicate P-values: * P<0.05, ** P<0.01, *** P<0.001. n.s.: not significant.

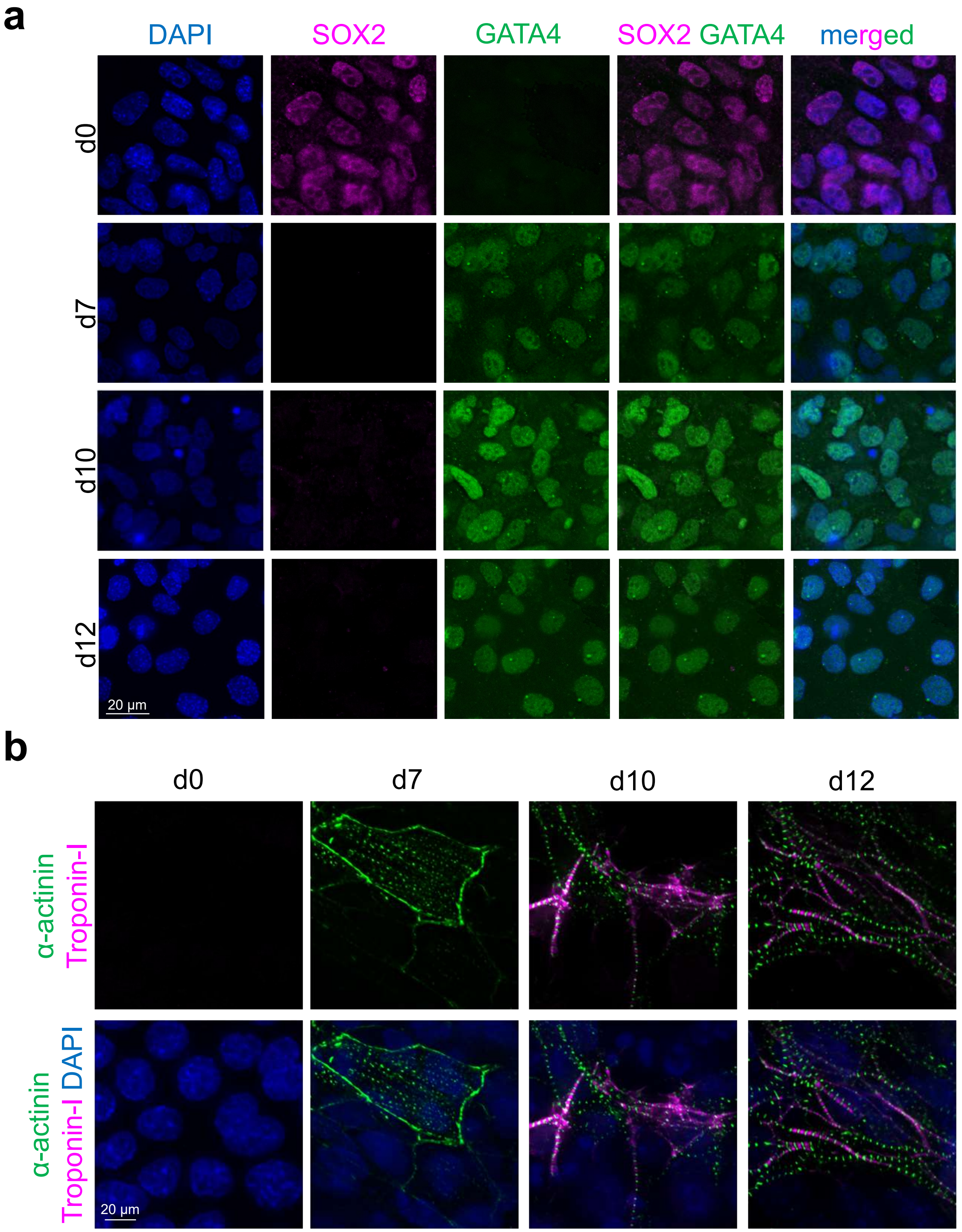

Figure S6. Cardiomyogenesis of P19CL6 cells. Immunofluorescence microscopy (IFM) analysis of cells after addition of DMSO. Samples were analysed at day 0 (d0), day 4 (d4), day 7 (d7), day 10 (d10) and day 12 (d12). **a.** IFM using antibodies against stem cell marker SOX2 (magenta) and the cardiac transcription factor GATA4 (green). **b.** IFM using antibodies against Troponin-I (magenta) and α-actinin (green) Nuclei were stained with DAPI (blue).

a

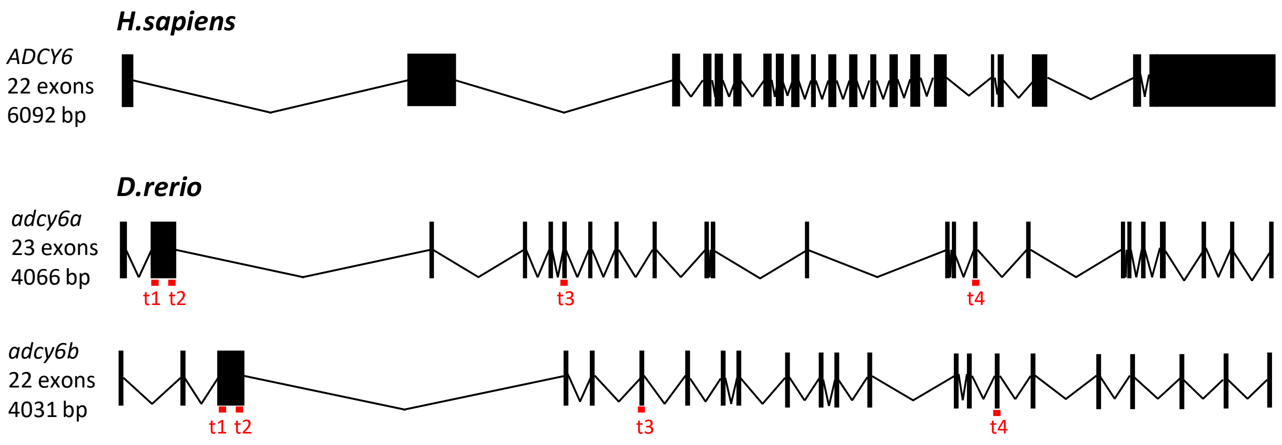

b

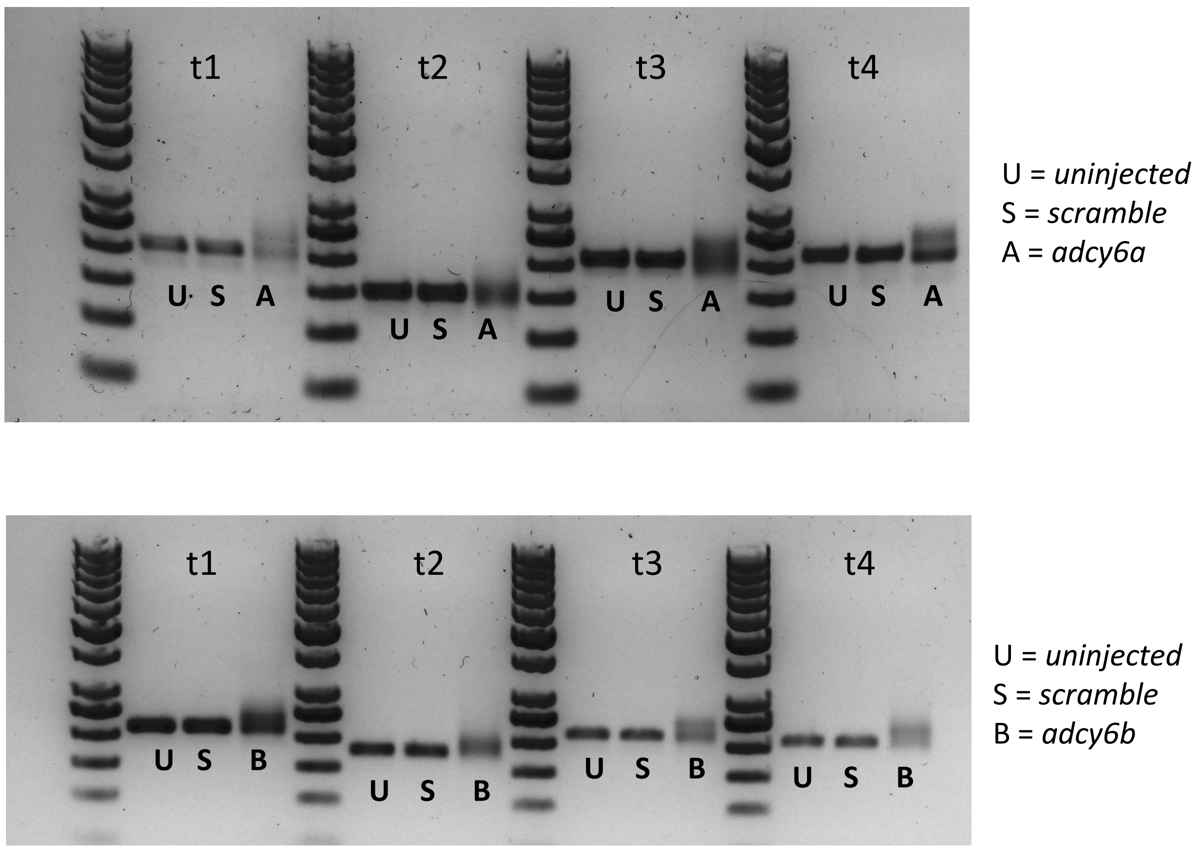

Figure S7. CRISPR-cas9 mediated mutagenesis of *adcy6* in F0 zebrafish embryos (crispants). **a.** Four guides were designed to target each of the zebrafish *adcy6a* and *adcy6b* gene, respectively. The zebrafish ortologues are shown below the human *ADCY6* gene. Exon sequence is indicated with black squares, intron sequence is indicated with lines. Target sites (t1-t4) of guide RNAs are shown with red lines. **b.** Example of genotyping gel for analysis of mutagenesis efficiency in crispants.

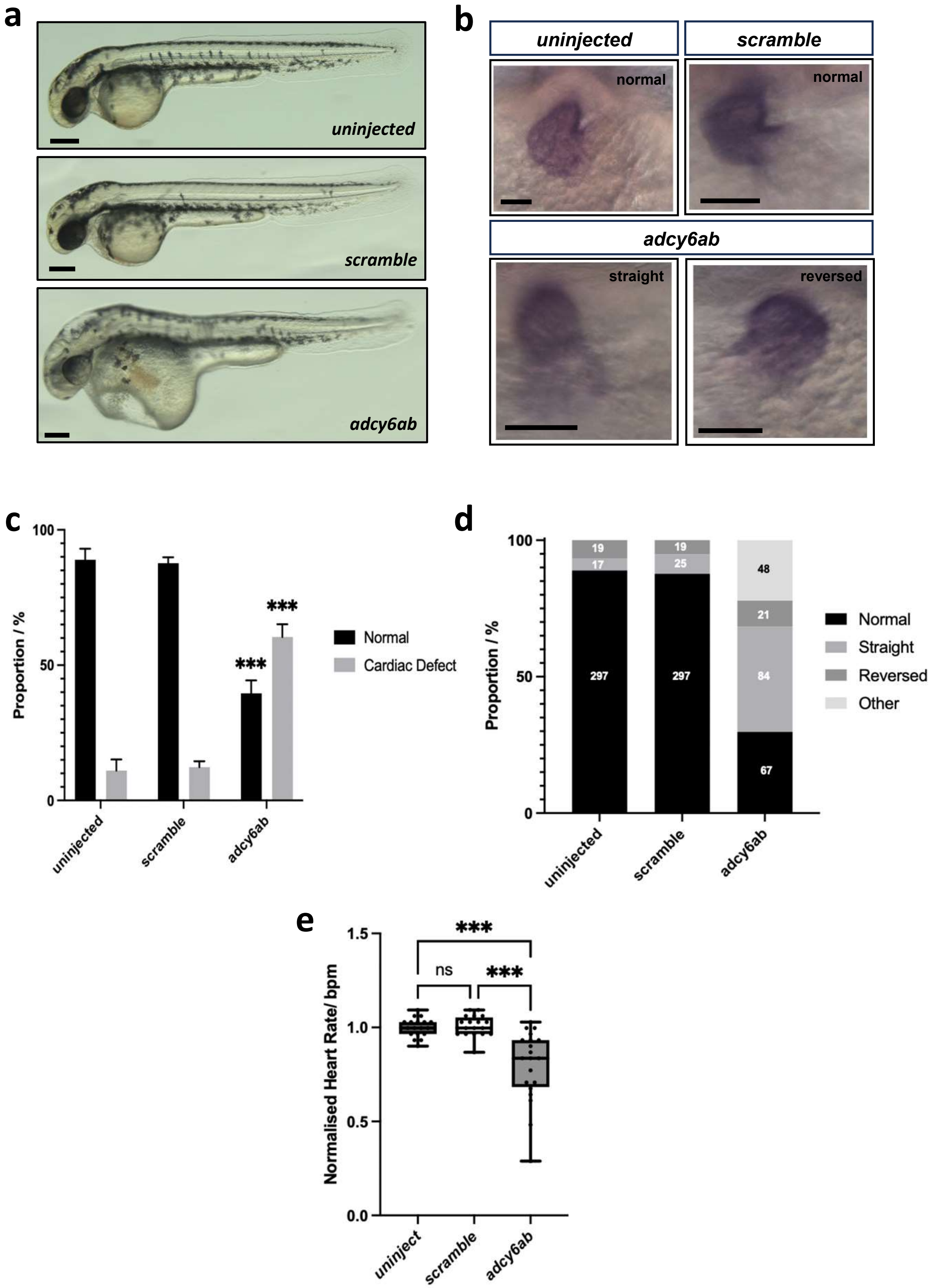

Figure S8. Double knockout of *adcy6a* and *adcy6b* causes heart defects **a.** Bright-field images showing the morphology of 2 dpf uninjected, scramble, and *adcy6ab* F0 crispant zebrafish larvae. Scale bars, 0.5 mm. **b.** mRNA expression of myl7 in 2 dpf crispant hearts. Upper panels show control larvae. Bottom panels show *adcy6ab* crispants. Scale bars, 100 µm. **c.** Proportions of cardiac defects observed in 2 dpf mRNA expression analysis of *myl7*. **d.** Proportion of cardiac phenotypes observed in 2 dpf F0 crispants. Numbers central within bars indicate number of larvae in each classification. **e.** Normalized heart rate measurements for 2 dpf F0 crispants in beats per minute (bpm). Two-way ANOVA (**c**) and ordinary one-way ANOVA (**e**) used for statistical analysis. Asterisk indicate P-values: *** P<0.001, n.s: not significant.

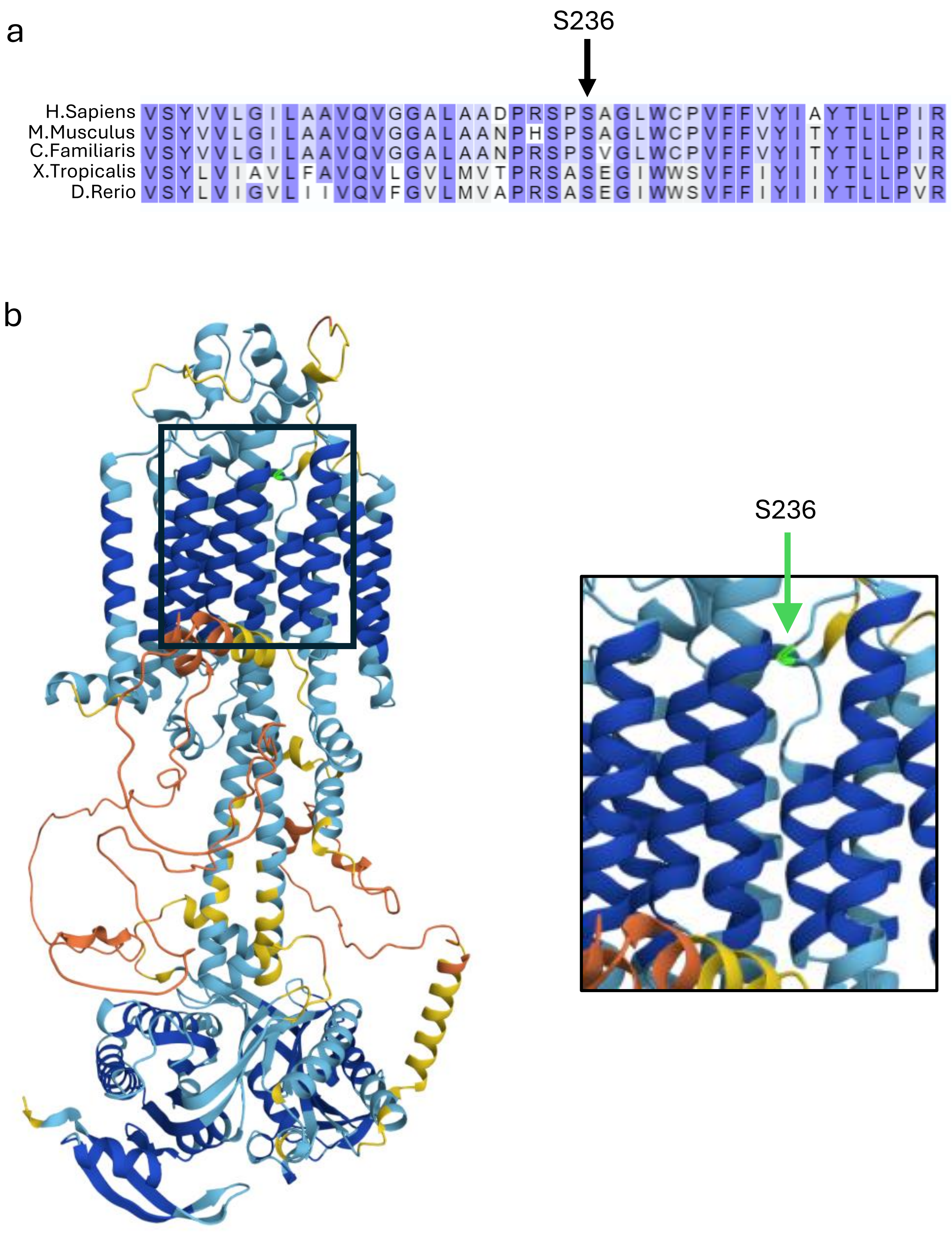

Figure S9. a. Sequence alignment of ADCY6 orthologues. **b.** Alphafold model of ADCY6. The position of S236 is indicated by arrows.
